# Applications of Nanobiomaterials in the Therapy and Imaging of Acute Liver Failure

**DOI:** 10.1007/s40820-020-00550-x

**Published:** 2020-11-19

**Authors:** Yuanyuan Jin, Haixia Wang, Ke Yi, Shixian Lv, Hanze Hu, Mingqiang Li, Yu Tao

**Affiliations:** 1 Laboratory of Biomaterials and Translational Medicine, The Third Affiliated Hospital, Sun Yat-sen University, Guangzhou, 510630 People’s Republic of China; 2 Department of Bioengineering, University of Washington, Seattle, WA 98195 USA; 3 Department of Biomedical Engineering, Columbia University, New York, NY 10027 USA

**Keywords:** Acute liver failure, Nanomaterials, Drug/gene/cytokines delivery, Targeted therapy, Imaging

## Abstract

**Highlights:**

- This review focuses on the therapeutic mechanisms, targeting strategies of various nanomaterials in acute liver failure, and recent advances of diverse nanomaterials for acute liver failure therapy, diagnosis, and imaging.
- This review provides an outlook on the applications of nanomaterials, especially on the new horizons in acute liver failure therapy, and inspires broader interests across various disciplines.

**Abstract:**

Acute liver failure (ALF), a fatal clinical disease featured with overwhelming hepatocyte necrosis, is a grand challenge in global health. However, a satisfactory therapeutic option for curing ALF is still absent, other than liver transplantation. Nanobiomaterials are currently being developed for the diagnosis and treatment of ALF. The liver can sequester most of nanoparticles from blood circulation, which becomes an intrinsic superiority for nanobiomaterials targeting hepatic diseases. Nanobiomaterials can enhance the bioavailability of free drugs, thereby significantly improving the therapeutic effects in ALF. Nanobiomaterials can also increase the liver accumulation of therapeutic agents and enable more effective targeting of the liver or specific liver cells. In addition, stimuli-responsive, optical, or magnetic nanomaterials exhibit great potential in the therapeutical, diagnostic, and imaging applications in ALF. Therefore, therapeutic agents in combination with nanobiomaterials increase the specificity of ALF therapy, diminish adverse systemic effects, and offer a multifunctional theranostic platform. Nanobiomaterial holds excellent significance and prospects in ALF theranostics. In this review, we summarize the therapeutic mechanisms and targeting strategies of various nanobiomaterials in ALF. We highlight recent developments of diverse nanomedicines for ALF therapy, diagnosis, and imaging. Furthermore, the challenges and future perspectives in the theranostics of ALF are also discussed.
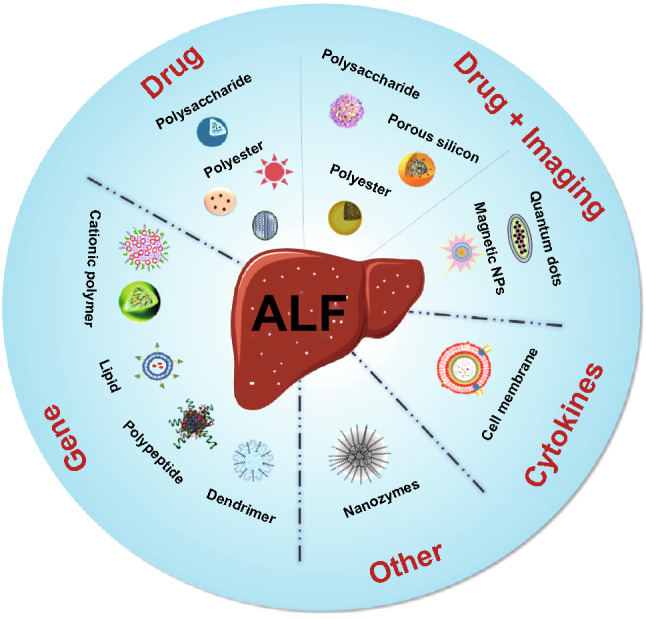

## Introduction

Acute liver failure (ALF) is a clinical syndrome with widespread hepatocellular necrosis, acute deterioration of liver function, and multiorgan dysfunction, inducing dreadful high mortality. ALF is usually derived from various pathogenesis, including viruses, drugs, poisonous substance, and other hereditary or autoimmune diseases [1, 2]. Liver transplantation is the only curative therapy so far, nevertheless, expensive costs, lack of donor livers, and complications associated with immunosuppression limit its application [3]. Therefore, alternative therapeutic strategies for ALF are greatly demanded.

Recently, promising progress in ALF therapy has been brought by the newly developed conventional drugs, such as small-molecule drugs, nucleic acids, and cytokines. However, they usually suffer from the disadvantages of poor solubility, low bioavailability, short half-life, easy degradation, or non-specific organ toxicity. Due to non-specific targeting and the first-pass effect [4], no sufficient amount of drugs can be specifically delivered to the liver, not to mention hepatocytes, macrophages, or other types of cells in the liver [5]. In addition, stem cell transplantation has also become an important alternative strategy for ALF therapy [6–8], whereas the lack of methods to monitor the distribution and survival rate of stem cells after transplantation [9], and the low efficiency of stem cell differentiation into functional hepatocytes remain major challenges for its clinical applications [10, 11]. These drawbacks restrict the further application of the current treatments in ALF.

Nanomaterial-based drug delivery systems possess outstanding advantages over free therapeutic agents in ALF treatment. Although the use of antioxidants or anti-inflammatory drugs becomes a trend in the treatment of ALF [12–14], only limited efficacy can be observed in clinical trials [15, 16]. The undesirable therapeutic effect has mainly been ascribed to low accumulation in the damaged liver, rapid drug clearance, difficulty in maintaining the therapy at adequate levels for a long time, and adverse reaction owing to supplementation overdose [17, 18]. Nanomaterials can improve pharmacological properties of small-molecule drug, including enhancing the aqueous solubility, protecting drugs from premature clearance by macrophages or kidneys, and improving the stability in blood and controlled release capability [19, 20]. Nanocarriers are also capable of protecting biologic drugs such as nucleic acids, growth factors from premature release, and degradation. Importantly, pathological stimulus-activatable nanomaterials, which respond to biochemical changes, such as reactive oxygen species (ROS) and pH, can preferentially target release drugs in the inflammatory site. The on-demand delivery system can improve drug efficacy and bioavailability for the treatment of ALF.

Furthermore, targeted nanoparticles for disease therapy or diagnosis is a popular concept [21]. Due to the biological filtration function of the liver, 30–99% nanoparticles by systemic administration are impeded in the liver. This phenomenon becomes an intrinsic superiority for nanomaterials targeting hepatic diseases. Nanobiomaterials are currently being developed for the diagnosis and treatment of ALF. Nanobiomaterials delivery systems can increase the accumulation of therapeutic agents in the liver. On the one hand, the liver has the natural advantage of capturing nanoparticles owing to its own structural characteristics. The liver receives two major sources of blood supply (hepatic artery and hepatic portal vein), with abundant blood flow. Approximately 1500 mL of blood flows through the adult liver every minute [22, 23]. Moreover, the liver contains the mononuclear phagocyte system with rich population of macrophages (Kupffer cells) that take up nanoparticles [21]. In addition, the extensive network of liver sinusoids in the liver is the largest reticuloendothelial cell meshwork, which slows down the blood flow within liver sinusoids [22, 24], and further increases the capture of nanoparticles. On the other hand, with unique size and surface characteristics, nanomaterial-based drug delivery systems determine its great superiority of preferential accumulation in liver through either passive or active pathways [25]. Nanoparticles modified with specific target ligands can even enhance specific penetration and uptake of cells, thus reducing off-target toxicity and side effect associated with undesired organ distribution [19, 20].

**Fig. 1:**
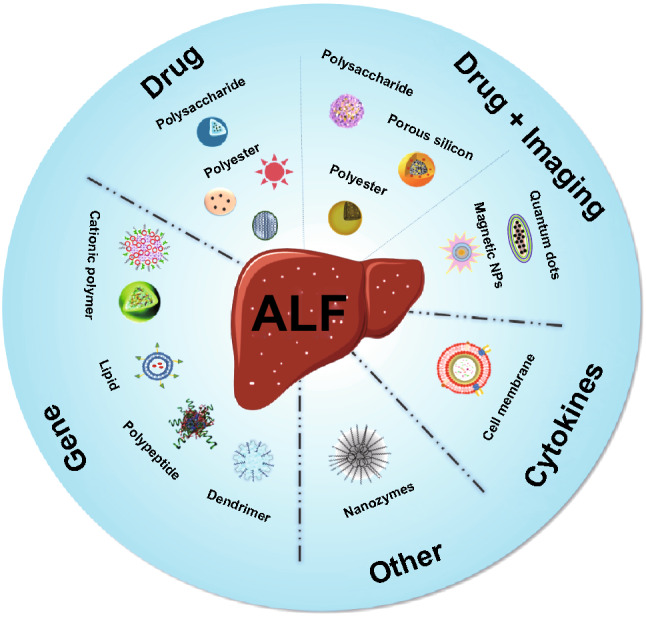
Schematic representation of diverse types of nanoparticles in the theranostics of ALF

Additionally, the novel nanovectors can be readily integrated with optical or magnetic imaging modalities to be utilized for cell or tissue imaging. These nanomaterials can label transplanted stem cells for assessing the cellular distribution and vitality, mark damaged areas for ALF diagnosis, or monitor treatment processes. Therefore, these well-designed nanocarriers can be applied as multifunctional platforms for diagnosis, treatment, and theranostics of ALF. So far, diverse types of nanomaterials are under investigation for ALF therapy, diagnosis or imaging, including polymeric nanoparticles, lipid-based nanoparticles, inorganic nanoparticles (metal/metal-oxide-based, and silicon-based), dendrimers, and cyclodextrin. In this review, we first address the mechanisms of nanomedicines for treating ALF. Second, we highlight recent advances of various nanomaterials for diagnosis, imaging and targeted therapy applications of ALF, and discuss the targeting strategies of nanomaterials in ALF therapy (Fig. 1). Finally, the current challenges and outlook on research directions in this field are discussed for future investigation.

## Mechanism of Nanomedicines for Acute Liver Failure Therapy

### Animal Models of Acute Liver Failure

Massive and rapid hepatocyte death, systemic inflammatory response syndrome (SIRS), and systemic immunosuppression are the main pathological mechanisms of ALF [26, 27]. The animal models of ALF mainly include surgical model and drug model. Surgical models are based on hepatectomy (partial or total), or devascularization or ischemia–reperfusion [28], which are used in the ALF model of mice [29], rats [30], rabbits [31], dogs [32], and pigs [33–35], even baboons [36]. The partial hepatectomy model is equivalent to liver cancer patients who have undergone the major hepatectomy. 95% hepatectomy (all lobes except the half of the caudate lobe are resected) is a rat model of ALF [37]. 90% liver resection (only the caudate is left) is the upper limit for a reproducible model of ALF in mice [29]. Joyeux et al. developed a hepatic failure model of dog by subtotal hepatectomy (partial hepatectomy). The left caudate and biliary tract of liver, which accounted for 3 to 6% of the total liver mass, were left in the surgery [32]. Fick and co-workers used a two-staged liver devascularization procedure to establish a surgical rabbit model of fulminant hepatic failure [31]. The first stage: portosystemic shunting, including the end-to-side portacaval shunt (ETS-PCS) and small-diameter side-to-side portacaval shunt (STS-PCS). The second-stage procedure: the hepatic artery ligation to create complete liver ischemia. In the ETS-PCS group, rabbits rapidly developed hepatic encephalopathy and ischemic liver necrosis within 48 h, with a high mortality, while the survival rate in STS-PCS group was improved due to a certain amount of liver blood flow being preserved. Zurab et al. established a large animal model for fulminant liver failure in baboons to evaluate the therapeutic efficacy of microencapsulated porcine hepatocytes. It was demonstrated that 75% hepatectomy and warm ischemia for 60 min resulted in a significant increase in the aspartate aminotransferase (AST), alanine aminotransferase (ALT) and total bilirubin levels, severe steatosis, and overwhelming hepatocyte necrosis within the first 10 days [36]. Total hepatectomy is an irreversible experimental animal model of ALF. Previous research performed the total hepatectomy in pig. During the operation, the liver was totally resected and the vena cava, portal vein, and a prosthetic graft were left, or the liver was resected from the vena cava and a portacaval shunt was performed. The pigs survived for 27 h after operation [38]. Knubben et al. tried to anastomose the vena cava and the portal of liver and performed anastomosis to the intrathoracic vena cava before total hepatectomy. Ultimately, the mean survival time of pigs was 51.2 ± 18.7 h [35]. Portacaval shunting, the hepatic artery ligation and/or the common bile duct and accessory hepatic vessels occlusion can achieve the complete devascularization in the liver [39, 40]. Complete devascularization has been used in the research of artificial and/or bioartificial hepatic support devices [41, 42] or the study of testing the therapeutic effects of N-acetylcysteine [43]. The research revealed that the molecular adsorbents recirculating system reduced extracellular brain ammonia in an ALF model of pig induced by portacaval anastomosis and hepatic artery ligation [44]. Although complete devascularization is parallel to the anhepatic state [45], devascularization seems to be superior for the study of ALF caused by ischemia and side effects of ischemia, whereas total hepatectomy is more suitable for the investigation on the treatment of bioartificial liver support systems in the anhepatic status. Partial devascularization refers to a portacaval shunt and then temporally graded clamping of the hepatic artery at a later stage [46]. It was reported that clamping the hepatic artery in pigs for 75 min led to 50% mortality and the median survival time of 22 h. In contrast, drug models are more widely used in nanomedicine for ALF treatment. Currently, the pathological process of ALF is mainly stimulated by experimental animal models resulted from hepatotoxic chemical drugs, such as acetaminophen (APAP), lipopolysaccharide (LPS), D-galactosamine (D-GalN), carbon tetrachloride (CCl_4_), thioacetamide, and concanavalin A [47, 48]. Because of the clinical relevance, the most widely used experimental model is the APAP-induced ALF, which mainly leads to massive necrosis of hepatocytes by causing mitochondrial dysfunction and nuclear DNA damage. 200–600 mg kg^−1^ APAP causes remarkable liver toxicity in fasted mice within 6 to 24 h following intraperitoneal injection or gavage [47, 49]. Lipopolysaccharide and D-galactosamine (LPS/D-GalN) are also common hepatotoxins that are widely applied to induce ALF in rat or mouse model [50]. Mice are intravenously injected with LPS/D-GalN via tail vein, the dose ratio varying in different studies [51, 52]. High dose of D-GalN exhausts the cellular uridine triphosphate and suppresses the synthesis of mRNA, leading to the massive diffuse hepatic apoptosis and necrosis. D-GalN is the most commonly used hepatotoxic agent in large animal models of ALF, in which D-GalN is administered directly through the jugular or central vein. 0.5–1.5 g kg^−1^ D-GalN can induce fulminant hepatic failure in pigs [8, 53, 54], while 0.2–0.3 g kg^−1^ D-GalN is used for constructing D-GalN-related cynomolgus monkey model of ALF [55, 56]. LPS triggers the activation of Kupffer cells and production of tumor necrosis factor-α (TNF-α), resulting in inflammatory necrosis of hepatocytes. Apoptosis and inflammation are recognized as features of LPS/D-GalN induced ALF [48]. Acute liver injury triggered by CCl_4_ is also a frequently used animal model of ALF. High doses (≥ 1 mL kg^−1^) of CCl_4_ can cause significant acute liver damage. Following intraperitoneal injection, CCl_4_ is converted to a radical (CCl_3_∙), which leads to mitochondrial damage and oxidative stress [47]. Thioacetamide-related ALF mice or rats models are usually established via intraperitoneal administration or gavage [57]. Thioacetamide shows obvious hepatotoxicity at doses (≥ 100 mg kg^−1^) owing to the metabolites (thioacetamide S-oxide and S, S-dioxide) [58]. In addition, the concanavalin A model is a typical and well-established experimental model for autoimmune hepatitis or viral hepatitis-mediated ALF [59, 60]. Concanavalin A induces T cell-mediated liver damage. 20 mg kg^−1^ concanavalin A through intravenous administration causes massive hepatic injury in mice within 8 to 10 h [61]. In general, hepatotoxic drug-induced ALF mouse or rat models are the most extensively used in nanomedicine.

### Mechanism of Acute Liver Failure Therapy Using Nanomedicines

Based on the aforementioned experimental animal models of ALF, the molecular mechanisms of ALF have been extensively studied in fundamental research. Studies have confirmed that hepatocellular damage is the pathological mark of hepatitis and a key driver for the development of hepatic diseases [62]. APAP overdose, toxic substances or hepatitis virus induced ALF is characterized by overwhelming hepatocyte death [63]. Massive hepatocyte death results in damage-associated molecular patterns (DAMPs) release [63]. Under the severe systemic infection, bacteria and other pathogens lead to the derivation of pathogen-associated molecular patterns (PAMPs) [27]. In return, largely increased DAMPs and PAMPs are recognized by pattern recognition receptors (PRRs), which activates the liver-resident Kupffer cells to secrete pro-inflammatory cytokines (e.g., TNF-α, interleukin-1 β (IL-1β), IL-6), ROS and chemokines, thereby amplifying pro-inflammatory signals. These pro-inflammatory factors accelerate the recruitment of bone marrow derived cells (mainly neutrophils and monocytes) into the liver promoting the inflammatory process [64, 65]. High levels of pro-inflammatory cytokines in circulation even lead to SIRS [66, 67]. Therefore, the degree and speed of hepatocyte necrosis, activation and infiltration of inflammatory bodies, and release of inflammatory factors are closely correlated to the occurrence and development of ALF [51]. Systemic immunosuppression plays another crucial role in the pathogenesis of ALF [27]. Excessive inflammatory response induces immoderate consumption of immune cells [68, 69], thus impairing innate and adaptive immune function [69–71], in other word, immunosuppression. Macrophages in the liver play a predominant role in the progression of inflammatory responses. A large number of monocytes derived from bone marrow are recruited into the liver following acute injury [72, 73]. With the deterioration of ALF and SIRS induced by monocyte infiltration and pro-inflammatory cytokines secretion [27], monocytes are functionally impaired and heavily depleted, which causes hyporesponsiveness to microbial challenge. These are hallmarks of immunosuppression in ALF [69, 70].

Hence, protecting hepatocyte, suppressing inflammatory storm, and regulating immunity play a critical role in the therapy of ALF. For one thing, according to mechanisms of the occurrence and development of ALF, current research indicates that protecting hepatocyte and suppressing inflammatory reaction are the main mechanisms of nanomedicines for treating ALF. Anti-inflammatory or antioxidant agents can be loaded in or conjugated with nanomaterials to target the injury site and liver cells, thereby relieving liver damage induced by inflammation and oxidative stress [12, 74–76]. Nanomaterials for delivery of small interfering RNA (siRNA) of pro-inflammatory factor to macrophage (Kupffer cell), specifically inhibit the secretion of pro-inflammatory cytokines and suppress massive inflammatory response [51, 77–79]. Moreover, drugs or genes with hepatoprotective functions are also transmitted to the liver by nanomaterials delivery systems to reduce apoptosis or necrosis of hepatocyte [80–83]. For another, nanotechnology is regarded as an assisted therapy strategy of stem cell transplantation to track the distribution and survival of transplanted cells [9]. Cell growth factors loaded nanoparticles can facilitate stem cell differentiation and promote liver regeneration by continuously releasing growth factors [10, 84, 85]. The applications of nanomaterials in the treatment of ALF will be described in detail below.

## Nanomedicine for Therapy of Acute Liver Failure

### Nanobiomaterials for Small-Molecule Drug Delivery

Clinical applications of some routine drugs in ALF therapy are limited because of their poor solubility, non-specific organ toxicity, and poor stability in blood circulation [12, 74, 76, 81, 86]. Nanobiomaterials can be designed as superior delivery systems with good hydrophilicity, blood system stability, and adequate positive surface charges to address these major clinical and pharmacological challenges of conventional drugs. Small-molecule drugs encapsulated in nanocarriers are generally more stable in blood circulation and more effective in treating disease than the free drug. Moreover, compared to classic pharmaceutical delivery systems (such as tablets or injectable solutions), nanomaterial-based drug carriers with unique particle size or ligand modification render the entire complex to be selectively and directly transmitted to the damaged site of liver even particular cells. Nanomedicines have favorable biodistribution and pharmacokinetic profile, which are conducive to reducing the incidence and severity of non-specific organ toxicity and improving therapeutic efficiency [87]. Based on the above characteristics, presently, various types of drug-loaded nanocarriers have been employed in the investigations of ALF therapy [88], which we will discuss in the following section.

Nanomaterials delivery systems can efficiently improve the water solubility of conventional drugs. Naringenin (4′,5,7-trihydroxyflavanone), a natural flavonoid aglycone of naringin, can scavenge free radicals via donating hydrogen to ROS [89, 90]. However, the insolubility in water hampers its clinical applications. The Eudragit^®^ E cationic copolymer is easy to be disrupted in the stomach, since its basic site of dimethylamino groups can be ionized in gastric juice [91]. Hence, Eudragit^®^ E cationic copolymer is commonly exploited to increase the solubility of hydrophobic drugs. The Eudragit^®^ E cationic copolymer was successfully utilized to load naringenin (naringenin-loaded nanoparticles system NARN), thereby forming nanoscaled particles (66.2 ± 0.38 nm). The crystal structure of naringenin was transformed to an amorphous state of NARN after synthesis. These changes of NARN further improved the solubility and the release of naringenin [76]. In simulated gastric medium, free naringenin (< 25%) was released within 120 min, whereas the release of naringenin from NARN was more than 95% during 20 min. The gastric solubility profile of NARN was beneficial to enhance the bioavailability of naringenin on oral administration. The naringenin-loaded nanoparticle was applied in the treatment of CCl_4_-induced ALF, which promoted the production of enzymatic antioxidants including catalase (CAT), glutathione peroxidase (GPX), and superoxide dismutase (SOD). Compared with the CCl_4_-intoxicated group, free naringenin and NARN group effectively reduced the levels of AST and ALT (*P* < 0.05), while the AST and ALT levels of NARN were 2 and 1.5 times lower than free naringenin, respectively. NARN manifested more robust hepatoprotective, antioxidant, and antiapoptotic pharmacological effects than naringenin.

Taking advantage of nanomaterials, the stability of drugs in the bloodstream can be improved as well. Poly(vanillyl alcohol-co-oxalate, PVAX) is a biodegradable and antioxidant polymeric prodrug, containing H_2_O_2_ responsive peroxalate ester bonds and vanillyl alcohol. Peroxalate ester bonds reacting with H_2_O_2_ can facilitate the hydrolytic degradation of PVAX. Accordingly, PVAX can remove H_2_O_2_ and promote the vanillyl alcohol (an anti-inflammatory and antioxidant ingredient) release [86]. Manganese porphyrin, with chemical versatility and catalase activity, is a non-peptidyl mimetic of superoxide dismutase [92]. However, it lacks the stability to circulate in the blood for a long time. Taking the superiorities of intrinsic antioxidant, anti-inflammatory, and rapid hydrolytic degradation capacities in PVAX, Ko and co-workers utilized PVAX to load manganese porphyrin. The release process of manganese porphyrin from PVAX particles in phosphate-buffered saline (PBS) lasted about 48 h and underwent two stages: rapid release and gradual release. The release mode not only improved the stability and perdurability of manganese porphyrin under physiological conditions, but also was suitable for the treatment of acute diseases. It was demonstrated that using PVAX for manganese porphyrin delivery exerted a synergistic effect in APAP-induced ALF compared to free drug. Manganese porphyrin-loaded PVAX particles effectively inhibited the generation of ROS and pro-inflammatory cytokines such as IL-6, iNOS, IL-1β, and COX-2, as well as significantly declined the ALT level (*P* < 0.001 relative to APAP group). Furthermore, due to the hydrodynamic diameter of ~ 2 μm, the particles could passively target to macrophages (or Kupffer cells) [12].

In addition, nanoparticles functionalized with a specific ligand or bioactive substance can target corresponding liver cells, increasing the specificity of therapy. Interleukin-1 receptor antagonist (IL-1Ra) connects with IL-1R competitively, therefore blocking the IL-1-induced intracellular signaling pathway and showing anti-inflammatory effect [93, 94]. Nevertheless, the application of IL-1Ra was limited owing to short biological half-life and high cost. Xiao and co-workers utilized lactosylated chitosan nanoparticle as the carrier of IL-1Ra. Chitosan nanoparticle possessed good biocompatibility and biodegradability. IL-1Ra released from the nanoparticles system mainly relying on the biodegradation and self-diffusion of chitosan. IL-1Ra chitosan nanoparticles had two quick release phases (~ 30%) and 30 h later entered a controlled release stage (~ 70%), which significantly prolonged the release and effect time of IL-1Ra. Moreover, lactosylated IL-1Ra chitosan nanoparticles manifested prominent hepatocytes targeting capacity via binding with the hepatocyte-specific asialoglycoprotein receptor (ASGP-R). Importantly, the combination treatment of IL-1Ra-loaded nanoparticles and transplanted mesenchymal stem cells (MSCs) exhibited synergistic effects in the therapy of ALF. The nanoparticles increased the survival rate of transplanted MSCs and suppressed the level of TNF-α, IL-1, and IL-6 (*P* < 0.05 compared with control group) [82]. Similarly, Roy et al. synthesized heparin-functionalized, andrographolide-loaded poly(lactic-co-glycolic acid) (PLGA) nanoparticles (Hep-AGnp) to increase the accumulation of andrographolide in the liver [81]. Although andrographolide has pronounced hepatoprotective activity, such as activation of antioxidant enzymes, membrane stabilization, and restoration of hepatic glutathione (GSH) levels, it shows inherently poor water solubility and permeability [95]. It was proved that the release pattern of andrographolide from andrographolide-loaded PLGA nanoparticles (AGnp) and Hep-AGnp nanoparticles was an initial rapid release phase followed by a slower and continuous release period. AGnp and Hep-AGnp nanoparticles were stable enough to show no aggregation within 3 months in refrigerated conditions. Besides, heparin possesses the ability to target Kupffer cells and endothelial-like cells lining the liver sinusoids. Heparin-coated gold nanoparticles were used as liver-specific computed tomography (CT) contrast agents [96]. Therefore, heparin-functionalized Hep-AGnp was capable of localizing rapidly in the liver, thereby upregulating GSH store and promoting antioxidant capacity in APAP-related liver injury. Compared with APAP-treated group (AST: 3519 ± 311.61 IU L^−1^, ALT: 4007 ± 383.50 IU L^−1^), the AGnp and Hep-AGnp nanoparticles groups significantly reduced the AST (576 ± 69.03 and 372 ± 32.53 IU L^−1^, respectively) (*P* < 0.001) and ALT (631 ± 77.22 and 406 ± 55.68 IU L^−1^, respectively) (*P* < 0.001) levels, while the GSH levels were close to normal (*P* < 0.001).

**Fig. 2:**
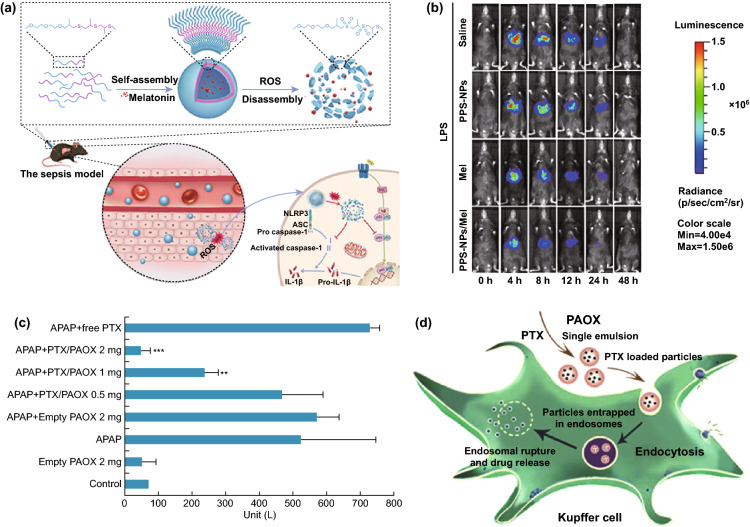
**a** Schematic illustration regarding the transmission and decomposition of melatonin-loaded poly(ethylene glycol)-b-poly(propylene sulfide) NPs (Mel-loaded mPEG-b-PPS-NPs), and the anti-inflammatory mechanism. **b** Bioluminescence imaging of hepatic dynamic NF-κB activation in NF-κB-Luc mouse models at various time points after LPS administered. (Compared with free Mel group, mice in the PPS-NPs/Mel group showed significantly lower luciferase signal.) Reprinted with permission from Ref. [74] **c** ALT level at different doses of PTX-loaded PAOX in APAP-induced ALF mice (mean ± SD, *n* = 4. **P < 0.01, ***P < 0.001 relative to APAP group). **d** Schematic representation of intracellular drug delivery of PTX-loaded PAOX particles (PTX: pentoxifylline, PAOX: poly(amino oxalate)). Reprinted with permission from Ref. [75]

Moreover, nanoparticles can further be designed to release agents in response to biochemical changes in the target microenvironments (such as ROS and pH). ROS is closely associated with the progression of inflammation-related diseases, in which the concentration of ROS in inflammatory cells is higher than normal cells [97]. In addition, an acidic extracellular microenvironment is one of the features of inflammatory site [98]. Thus, ROS or pH responsive nanomaterials may serve as an ideal delivery system to target the injured site. Biologically responsive polymeric nanoparticles have been widely exploited as a trigger or biological switch in drug delivery systems [99]. Melatonin is an endogenous free radical scavenger and antioxidant agent [100]. Studies have confirmed that melatonin can ameliorate sepsis-related tissue injury through suppressing the activation of nuclear factor κB (NF-κB) and the NLRP3 inflammasome, and protecting mitochondria [101, 102]. However, melatonin is often designed as an oral formulation with low bioavailability due to its short half-life (*t*_1/2_ < 30 min) [103]. Chen and co-workers synthesized methoxy poly(ethylene glycol)-b-poly(propylene sulfide), a ROS-responsive polymeric NPs, to serve as a drug vehicle for melatonin release (Fig. 2a, b). The polymeric NPs combined with melatonin through self-assembly and efficient encapsulation. Upon exposure to ROS, the hydrophobic sulfide moieties of the poly(propylene sulfide) block were converted to the more hydrophilic sulfoxides and sulfones [104, 105], resulting in the swelling of the NPs and the discharge of melatonin. About 81.78% melatonin was constantly released within 12 h in the presence of 10 mM H_2_O_2_. On the contrary, melatonin was tightly encapsulated in the physiological conditions. Compared with previous studies, the polymeric NPs were able to target the injured site and obviously prolong the biological half-life and bioavailability of melatonin [103, 106]. Melatonin-loaded NPs alleviated oxidative stress and hepatic injury via impeding NF-κB activation and the NLRP3 inflammasome/IL-1b pathway, as well as reducing levels of COX-2 and iNOS inflammatory mediators (*P* < 0.05 compared with the free melatonin group) [74]. The nanocomposites provided a sustained, on-demand, and targeted release of melatonin, which were much more efficient than free melatonin in sepsis-induced acute liver injury mice model.

On account of the size of nanoparticles, nanoscaled drug delivery systems possess the instinctive trend to target mononuclear phagocyte systems. Particles with a diameter in the range of 0.5–3.0 μm are readily phagocytized by macrophages, particularly macrophages of liver and spleen [107–109]. Kim et al. developed submicron poly(amino oxalate) (PAOX) particles for pentoxifylline (PTX) delivery in ALF therapy (Fig. 2c, d) [75]. PAOX was a fully biodegradable drug delivery system. PTX presented therapeutic effects on cirrhosis and acute alcoholic hepatitis [110]. PAOX particles not only possessed excellent biocompatibility, but also achieved endosomal escape through colloid osmotic pressure and proton sponge effects. It was demonstrated that PTX-loaded PAOX particles could passively target the liver and stimulate the uptake of macrophages owing to submicron scale structure. Additionally, the nanocomposites released the encapsulated PTX into the cytoplasm by inducing a colloid osmotic disruption of the phagosome. In vitro experiments confirmed that 45% of PTX was released instantly at pH 7.4 during the first 4 h. The total release amount reached 80% within 12 h, which suggested that PTX-loaded PAOX particles were suitable for acute inflammatory diseases. As a consequence, the particles decreased the level of ALT in a dose-dependent manner and alleviated liver cell injury in APAP-associated ALF. The therapeutic effect of PTX/PAOX group was significantly higher than the same dose of free PTX.

Based on the above research, at present, nanomaterials for small-molecule drug delivery are biodegradable biopolymer materials, with good biocompatibility. Antioxidative or anti-inflammatory drugs with poor water-solubility, short half-life, and low bioavailability can be combined with nanomaterials to form new nano-formulations, thereby improving the liver accumulation and bioavailability of small-molecule drugs. This new nano-formulation is expected to be a new therapeutic strategy for ALF. These studies evaluate the cellular compatibility in vitro and the biodistribution in major organs of nanomaterials and show that small-molecule drug delivery nanosystems are largely accumulated in macrophage-enriched organs, such as liver, spleen, and lung, with negligible cytotoxicity. However, there is no strict evaluation on pharmacokinetics of the nano-formulation in vivo. More research should be performed to achieve a deeper understanding of the fate and metabolic pathways of these nanomaterials in the body. In addition, what is the elimination pathway of degradation products of nanosystems in vivo? The kidney, the mononuclear phagocyte system or the hepatobiliary [111, 112]? There is no relevant data to prove it.

### Nanobiomaterials for Gene Delivery

#### Nanobiomaterial-Mediated RNA Interference

As mentioned before, liver inflammation in ALF is mainly initiated by DAMPs and PAMPs which leads to the activation of monocyte or macrophage, and promotes the secretions of pro-inflammatory cytokines, ROS and chemotactic factors. Afterward, the recruitment of inflammatory cells such as neutrophils and monocytes increases. These processes in turn amplify the inflammatory process, ultimately resulting in SIRS [64, 65]. Hence, anti-inflammation plays a vital role in the therapy of ALF [113]. RNA interference (RNAi)-mediated gene silencing is an important therapeutic method in inflammation-related diseases [78, 114, 115]. siRNA, microRNA (miRNA), and short hairpin RNA (shRNA) are usually exploited to downregulate specific cellular signaling molecules, including cytokines and chemokines [116]. siRNA, a double-stranded RNA molecule, binds to and degrades the complementary mRNA through RNA-induced silencing complex (RISC), specifically inhibiting gene expression [117]. siRNA is a crucial tool to silence genes of inflammatory cytokines [118–120]. However, naked siRNA is not stable in serum as it is readily digested by nucleases. In addition, naked siRNA is difficult to penetrate the cell membrane and form RISC in the cytoplasm due to surface negative charge [83, 121]. Nanotechnology-based non-viral gene delivery can address these hurdles. Nanobiomaterials-based siRNA delivery systems have been employed to achieve targeted gene silencing and improve the efficacy of gene therapy in ALF treatment.

**Fig. 3:**
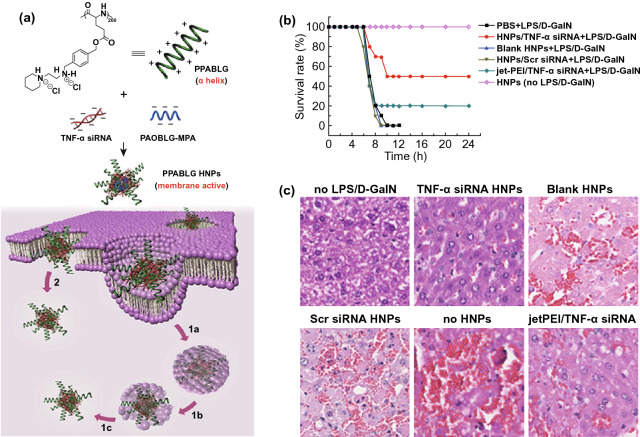
**a** Schematic illustration depicting the synthesis, effective cellular internalization, and endosomal escape processes of hybrid nanoparticulate (HNP) system based on a cationic helical polypeptide PPABLG (1a. Endocytosis; 1b. Pore formation on endosomal membrane; 1c. endosomal escape; 2. Direct transduction via pore formation on cell membranes). **b**, **c** Intravenous injection of the HNPs/TNF-α siRNA composites mediates anti-inflammatory effect in LPS/D-GalN-induced ALF. **b** The survival rate of mice was evaluated within 24 h following LPS/D-GalN stimulation (50 μg siRNA kg^−1^); **c** HE staining of liver sections in mice model of LPS/D-GalN induced ALF (PPABLG HNPs: PPABLG (a cationic helical polypeptide)/PAOBLG-MPA (an anionic polypeptide)/TNF-α; jet PEI: a commercially available gene delivery vector). Reprinted with permission from Ref. [78]

TNF-α plays a key role in the inflammation process [122], as well as the development of ALF. The level of TNF-α is significantly positively correlated with the severity of ALF [113]. He et al. designed a cationic helical polypeptide, a hybrid nanoparticulate system (HNP), which was an effective TNF-α siRNA delivery platform and showed the advantages of avoiding endosomal encapsulation and lysosomal degradation of TNF-α siRNA (Fig. 3) [78]. The cationic helical polypeptide induced pores formation on the cytomembrane and endosomal membrane, which facilitated the cellular uptake and endosome escape of TNF-α siRNA in macrophages. Consequently, the HNPs/TNF-α siRNA efficiently induced nearly 90% knockdown of TNF-α in LPS-stimulated RAW 264.7 cells and LPS/D-GalN-induced ALF mice model (*P* < 0.01 compared with the naked TNF-α siRNA group). The nanocomposites with 50 μg kg^−1^ TNF-α siRNA ultimately increased survival rate (50%) of mice compared with the commercially available gene delivery system (20%). Moreover, HE-staining revealed that the HNPs/TNF-α siRNA group had fewer inflammatory cells infiltration and injured hepatocytes than the free TNF-α siRNA group did. Similarly, TNF-α siRNA could also be delivered into macrophages using Se-PEI as the nanocarrier, to alleviate hepatic inflammation of ALF caused by LPS/D-GalN [51]. Se-PEI, 600 Da polyethyleneimine (PEI) cross-linked with diselenide bond, served as a delivery vector of TNF-α siRNA. The resulting polymer was further coated with carboxylated mannan (Man-COOH) via electrostatic interaction, which actively targeted mannose receptors on macrophages membrane and facilitated endocytosis. Interestingly, the diselenide bond was decomposed by ROS in inflammatory macrophages, which successively induced the degradation of Se-PEI, thereby releasing TNF-α siRNA. Specific targeting of nanomedicine could efficiently minimizes off-target toxicity. As a result, Man-COOH/Se-PEI/TNF-α siRNA nanocomposites remarkably decreased TNF-α levels in serum and liver tissues and exhibited promising anti-inflammatory potential in ALF.

Apart from TNF-α, Fas gene-mediated apoptosis also plays a pivotal role in viral or autoimmune hepatitis and alcoholic liver disease. Fas is highly expressed on hepatocytes, Fas-mediated apoptosis leading to hepatic injury [123]. Jiang et al. fabricated the galactose-conjugated liposome nanoparticles (Gal-Lipo NPs) for the transfer of Fas siRNA in fulminant hepatitis. The nanoparticle exhibited much higher stability than naked siRNA in serum. Furthermore, it was a hepatocyte-specific delivery system that could be specifically recognized by ASGP-R on hepatocytes. After systemic administration, Gal-Lipo NPs/siRNA complex significantly reduced the expression of Fas and the serum levels of ALT and AST in concanavalin A-induced fulminant hepatitis [83].

**Fig. 4:**
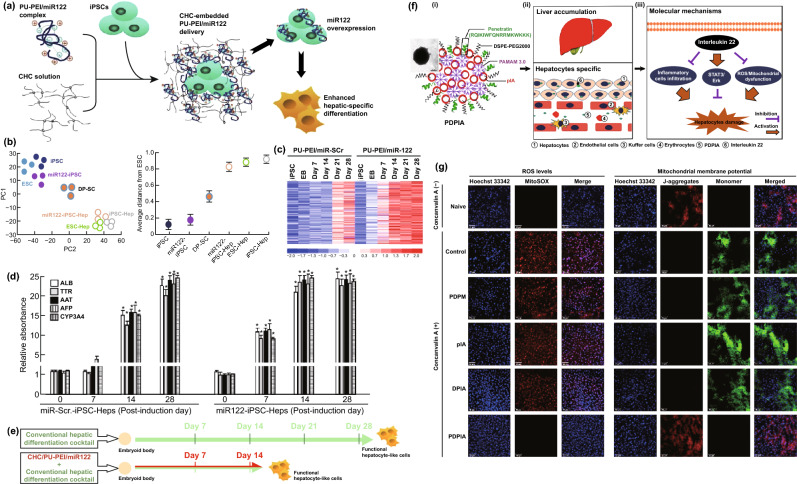
**a** Schematic representation about embedment of PU-PEI/miR122 complex in CHC solution, miR122 overexpression, and the enhanced hepatic differentiation. **b** Gene expression pattern of miR122-iPSC-Heps (multidimensional scaling analysis) (ESC: human embryonic stem cell; DP-iPSCs: dental pulp-derived induced pluripotent stem cells; miR122-iPSCs: stable miR122-overexpressing iPSCs; DP-SC: dentalpulp-derived stromal cell; ESC-Hep: ESC-derived hepatocyte-like cell; iPSC-Hep: iPSCs-derived hepatocyte-like cell). **c** Microarray results of gene expression profiles over differentiation course (miR-Scr.-iPSC-Heps vs. miR122-iPSC-Heps). **d** Hepatic-specific genes expression of miR-Scr.-iPSC-Heps and miR122-iPSC-Heps during differentiation process (quantitative RT-PCR) (ALB: albumin, TTR: transthyretin, AAT: a-antitrypsin, AFP: alphafetoprotein, CYP3A4: the liver enzyme cytochrome P450 3A4) (*P < 0.05 vs. post-induction day D0 in iPSC-Heps with corresponding treatment). **e** Schematic illustration depicting the CHC/PU-PEI-mediated miR122 delivery shortens the period of hepatic induction. Reprinted with permission from Ref. [84]. **f** i) Composition of polypeptide penetratin-based hybrid nanoparticles (PDPIA) (DSPE-PEG2000: PEGylated lipids; PAMAM 3.0: polyamidoamine dendrimer; pIA: the IL22 expression plasmid), ii) Liver accumulation and predominant hepatocyte internalization process of PDPIA, iii) Anti-inflammatory mechanism in amelioration of concanavalin A-induced hepatitis. **g** ROS accumulation (Mitosox stain) and the mitochondrial membrane potential (JC-1 stain) in liver tissues were analyzed by confocal microscope (red fluorescence: ROS/normal membrane potential; green fluorescence: abnormal membrane potential; blue fluorescence: nuclei (Hoechst 33342); PDPM: penetratin-based hybrid nanoparticle system, containing 50 μg empty plasmid; DPIA: hybrid nanoparticle system without penetratin). Reprinted with permission from Ref. [80]

In addition, MicroRNA122 (miR122) is a liver-specific microRNA, which is highly associated with the differentiation of matured hepatocytes and hepatocyte metabolism regulation [124]. Overexpression of miR122 can facilitate stem cells to differentiate into matured hepatocytes [125–127]. To achieve efficient miR122 delivery, miR122 was conjugated to the biodegradable polyurethane-graft-short-branch polyethylenimine (PU-PEI) (Fig. 4a–e) [84]. The copolymer (PU-PEI/miR122) was further embedded into nanostructured amphiphatic carboxymethyl–hexanoyl chitosan (CHC/PU-PEI/miR122). This nanoscaled delivery system successfully delivered miR122 to human dental pulp-derived induced pluripotent stem cells (DP-iPSCs) and improved transfection efficiency of miR122 (~ 40% of lentiviral-mediated transfection efficiency). (Lipofectamine 2000 and PU-PEI/miR122-mediated efficiencies were ~ 19% and ~ 26% of lentiviral-mediated transfection efficiency, respectively.) Remarkably, the expression of miR122 in CHC/PU-PEI/miR122 group increased by nearly eightfold compared with the PU-PEI/miR122 group. As a result, CHC/PU-PEI/miR122 effectively promoted the hepatic-specific differentiation of DP-iPSCs into functional hepatocyte-like cells (miR122-iPSC-Heps). The multidimensional scaling analysis revealed that the gene expression profile of miR122-iPSC-Heps was close to the gene signatures of DP-iPSCs-derived hepatocyte-like cells (DP-iPSC-Heps) and human embryonic stem cell-derived hepatocyte-like cells (ESC-Heps) (Fig. 4b). Hepatic-specific genes in miR122-iPSC-Heps were upregulated and achieved maximal expression after ~ 14 days post-differentiation (Fig. 4c, d), which illustrated that CHC/PU-PEI/miR122 nanocomplexes reduced the hepatic induction period to 14 days (Fig. 4e). Furthermore, the transfected DP-iPSCs preserved liver-specific functions of hepatocytes such as albumin secretion and showed hepatoprotective efficacy in thioacetamide-induced ALF therapy in vivo.

As NF-κB is a regulator in the inflammatory reaction, preventing NF-κB activation of macrophages to treat liver failure is promising. The double-stranded oligonucleotide NF-κB decoy contains NF-κB binding sequences, which can efficiently suppress NF-κB-mediated transcription via connecting with activated NF-κB [128–130]. Ogushi’s group used HVJ liposomes (fusogenic liposomes with hemagglutinating virus of Japan) to carry NF-κB decoy to attenuate inflammatory cytokine production in LPS-induced ALF [131]. In a similar study, Hoffmann and co-workers employed gelatin nanoparticles to load NF-κB decoy. The nanocarriers enhanced the uptake efficiency of NF-κB decoy in Kupffer cells, which consequently prolonged survival time of LPS/D-GalN-induced liver injury mice model [132]. Despite the satisfactory therapeutic effects, HVJ liposomes and gelatin nanoparticles target to Kupffer cells in a non-specific manner [133, 134], leading to low accumulation. In order to selectively restrain NF-κB activation in Kupffer cells without interfering with hepatocytes, Higuchi et al. designed mannosylated cationic liposomes (Man-liposomes) as vectors for NF-κB decoy delivery [79]. The specific endocytosis of Man-liposomes by Kupffer cells was achieved via recognizing the mannose receptor. Compared with this polyplex, the liver accumulation of NF-κB decoy delivered by bare cationic liposomes was much lower. Moreover, when an equal amount of NF-κB decoy was loaded, cationic liposome/NF-κB decoy nanocomposites without mannosylation could not inhibit the production of cytokine. NF-κB decoy-loaded man-liposomes specifically inhibited the production of interferon γ (IFN γ), TNF-α, and IL-1β and significantly decreased the levels of ALT and AST, thereby mitigating hepatic impairment in LPS-intoxicated ALF mice models. Taking advantage of specific fucose receptor of Kupffer cells, Akao and co-workers developed fucose-modified dendrimer/α-cyclodextrin conjugates to selectively transfer NF-κB decoy to the liver. The nanocomplex was intravenously injected into mice to cure LPS-related fulminant hepatitis, which resulted in not only suppressing the production of nitric oxide and TNF-α in LPS-stimulated NR8383 cells, but also attenuating the levels of TNF-α, AST, and ALT in vivo, consequently, enhancing the survival rate of mice model [77].

#### Nanobiomaterial-Mediated DNA Therapeutics

Aside from RNAi therapeutic delivery, DNA-based gene therapy is also a promising approach in ALF therapy. Interleukin-22 (IL-22) is an effective survival factor of hepatocyte, which protects hepatocytes and repairs hepatic damage through activating the signal transducer and activator of transcription 3 (STAT3)/extracellular signal regulated kinase (Erk) signaling transduction [135]. Local expression of IL-22 is a promising strategy to alleviate hepatocellular injury in the therapy of severe hepatitis [80]. However, owing to the widely expressed IL-22 receptor, systemic intravenous administration of IL-22 causes non-specific side effects, such as multiple sclerosis, rheumatoid arthritis, and so on [136]. Clearance by mononuclear phagocyte system, low transmit efficiency and endosomal/lysosomal entrapment are also the difficulties that need to be dealt with during IL-22 gene delivery [137]. Chen et al. constructed a self-assembling polypeptide penetratin-based hybrid nano-formulation composed of polyamidoamine 3.0 (PAMAM 3.0) dendrimer, PEGylated lipids, penetratin peptides and IL-22 expression plasmid to deliver and locally express IL-22 (Fig. 4f, g) [80]. In combination with penetratin and polyamidoamine dendrimers, the nanocomplex showed distinct merits including penetratin-mediated cellular even endosomal membrane penetration, endosomal escape, almost no uptake of Kupffer cells, in particular, predominant hepatocyte internalization. The penetratin-based hybrid nano-formulation preferentially accumulated and locally expressed IL-22 in hepatocytes after systemic administration. It activated STAT3/Erk signal transduction and thus promoted hepatocyte regeneration, efficiently inhibited ROS accumulation and protected mitochondrial function, exhibiting robust anti-inflammatory efficacy in mice model of concanavalin A-induced severe hepatitis.

In another example, Xu and co-workers constructed a multi-combination nanocomposite that was composed of Fe@Au, fisetin, inactive rhomboid protein 2 siRNA, and TNF-α inhibitor. Fisetin, a natural antioxidant, is found in various vegetables and fruits [138, 139]. TNF-α inhibitor manifested excellent anti-inflammatory and antioxidant effects in Listeria monocytogenes induced fatal septicemia-associated ALF [140]. The nanocomposite inhibited macrophage activation-related inactive rhomboid protein 2/TNF-α converting enzyme/TNF-α signaling and restored nuclear factor erythroid-2-related factor 2 to relieve Listeria monocytogenes-induced metabolic disorder and septicemia-associated systemic inflammation.

Gene delivery to the liver is a complex process, including transportation in blood circulation, accumulation in the liver, liver cells internalization through membrane fusion or endocytosis, and gene release in the cytosol or nucleus [141]. Gene delivery provides an exciting approach for the therapy of different diseases [142–145], as well as the anti-inflammatory treatment of ALF. However, gene delivery has been hampered by the premature degradation, endosomal/lysosomal entrapment, undesired biodistribution, and minimal intracellular delivery [146]. Current studies show that nanomaterials such as cationic polymers (PEI [51], PAMAM [80]), and liposomes have been used as gene delivery systems in ALF treatment, due to the nucleic acids protection and excellent endosomal/lysosomal escape capability. Moreover, the delivery system increases accumulation of therapeutic agents in the liver, even the internalization of Kupffer cells, thereby limiting undesirable organ toxicities [51, 77, 80, 84, 143]. The side effects and systemic toxicity are major obstacles of the application of non-viral gene nanocarrier complexes. Most of the current studies only assess the cytotoxicity and biological distribution of the nanosystem. It is reported that the nanosystem is mainly gathered in macrophage-enriched organs (liver, spleen, and lung), with negligible cytotoxicity [51, 77, 79, 84]. A few studies have comprehensively assessed the biosafety and organ toxicity of materials by serological marker detection and histological analysis [78, 80]. It is worth noting that part of cationic nanoparticles is readily accumulated in the lung after systemic administration, which may be caused by the entrapment of lung vascular tree [147]. However, there is no morphological damage to major organs [80]. Meanwhile, the sub-organ or cell biodistribution of gene carriers needs to be further analyzed to explore the precise uptake mechanisms. Notably, the absence of pharmacokinetics is a common defect in all the studies.

### Nanobiomaterials for Cytokines Delivery

**Fig. 5:**
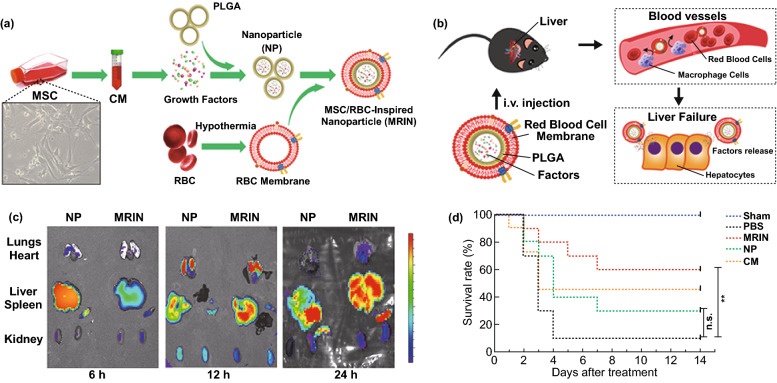
**a** Schematic representation about the synthetic process of MRINs: MSC-conditioned media (MSC-CM) preparation; MSC-CM being encapsulated in PLGA to form nanoparticles (NPs); fabrication of membrane vesicles from RBCs and cloaking of RBC membranes on NPs to form MSC/RBC-inspired nanoparticles (MRINs). **b** Intracellular kinetics of MRINs in ALF therapy. **c** Distributions of NPs (without RBC membrane) and MRINs at different time after intravenous injection in ALF mice model. **d** Survival rates of CCl_4_-induced ALF mice models (**P < 0.01 MRIN vs PBS) (Sham: health mice and no therapy; PBS: CCl_4_-induced ALF + 200 μL PBS tail vein injection; MRIN: 1 × 10^9^ MRINs in 200 μL tail injection (twice a week for 2 weeks); NP: CCl_4_-induced ALF + 1 × 10^9^ NPs (without RBC membrane) in 200 μL PBS tail vein injection; CM: CCl_4_-induced ALF + 1 mg conditioned media lyophilized powder dissolved in 200 μL PBS tail vein injection) Reprinted with permission from Ref. [85]

Stem cell transplantation is an alternative therapeutic strategy for liver transplantation in ALF [6–8]. Among them, MSCs transplantation is the most widely used method. Its mechanism in the treatment of ALF is still controversial. Some studies suggested that the therapeutic efficacy of stem cells was due to the regulation of inflammation by secreted cytokines and exosomes [148–152]. The therapeutic effects of MSC-conditioned medium for ALF could be the same as MSC transplantation. Liang et al. applied the MSC-conditioned medium incorporated PLGA nanoparticles for ALF therapy. The nanoparticles were further coated with red blood cell membranes (Fig. 5), constituting a stable cytokine delivery system. Biomembrane endowed the NPs with good biocompatibility and blood stability and discouraged endocytosis by the macrophage. Following intravenous injection, the nanoparticles escalated liver accumulation with time. Furthermore, it significantly reduced the level of pro-inflammatory factors, promoted liver regeneration, protected hepatic function, and enhanced survival rate in mice model of CCl_4_-induced liver failure [85].

**Fig. 6:**
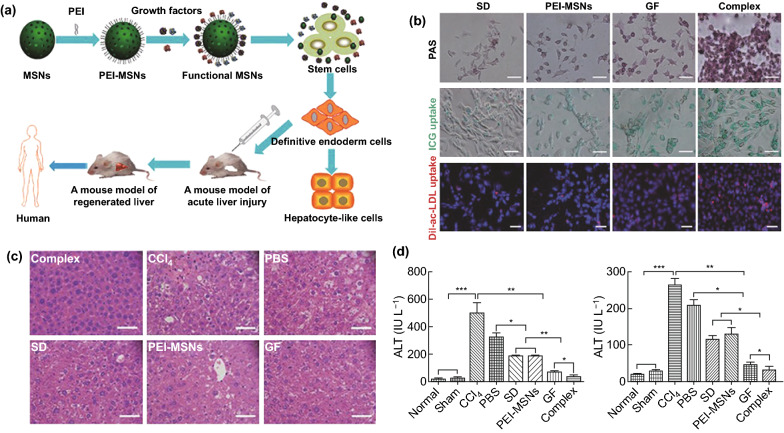
**a** Schematic illustration of growth factors loaded polyethyleneimine (PEI)-modified mesoporous silica nanoparticles (MSNs) (Functional MSNs complex GF-PEI-MSNs) for facilitating mouse embryonic stem cells to differentiate into hepatocyte-like cells. **b** Functional detections of hepatocyte-like cells differentiated from mouse embryonic stem cells (SD: embryonic stem cells without treatment; PEI-MSNs: polyethyleneimine-modified mesoporous silica nanoparticles; GF: growth factors alone, Complex: growth factors loaded polyethyleneimine-modified mesoporous silica nanoparticles (GF-PEI-MSNs); PAS: glycogen storage tested by periodic acid-Schiff staining, positive cells with a pink or red–purple cytoplasm; ICG uptake: the function of uptake measured by indocyanine green; LDL uptake: Dil-labeled acetylated low density lipoprotein). **c** HE staining of liver tissues among different groups. **d** Serum level of ALT and AST in diverse groups (mean ± SD (*n* = 3). * *P* < 0.05, ** *P* < 0.01, and *** *P* < 0.001. Normal: normal mice; Sham: corn oil administration alone; CCl_4_: CCl_4_-induced ALF; PBS: CCl_4_-induced ALF + 0.1 mL PBS injection; SD: CCl_4_-induced ALF + spontaneously differentiated mESCs; PEI-MSNs: CCl_4_-induced ALF + differentiated mESCs treated with PEI-MSNs alone; GF: CCl_4_-induced ALF + differentiated mESCs treated with growth factors alone; Complex: CCl_4_-induced ALF + differentiated mESCs treated with GF-PEI-MSN complexes. Reprinted with permission from Ref. [10]

Another mechanism of MSCs in ALF therapy is that MSCs can differentiate into hepatocyte-like cells in vitro and in vivo [153]. Hence, stem cell-derived hepatocyte transplantation is a potential therapy in ALF. However, a low rate of stem cell differentiation is the considerable obstacle to its application [10]. Previous studies reported that MSCs from bone marrow, amniotic fluid, umbilical cord, or adipose tissue could differentiate into hepatocyte-like cells via culturing in medium supplemented with some growth factors, such as basic fibroblast growth factor (bFGF), epidermal growth factor (EGF), or hepatocyte growth factor (HGF) [154–157], whereas the short half-life and poor stability of growth factors, and their cell toxicity at higher concentrations limit their biomedical applications [158]. Therefore, developing a sustained delivery system of growth factors is necessary to address these challenges. Wang et al. assembled HGF, acidic fibroblast growth factor (aFGF), and activin A into polyethyleneimine (PEI)-modified SiO_2_ NPs (MSN) to form the GF-PEI-MSN complex, an efficient delivery system of growth factors (Fig. 6). The complex was used to promote a direct differentiation of stem cells toward hepatocyte-like cells. The ability of glycogen synthesis and storage (periodic acid-Schiff (PAS) staining) and the cellular uptake capability of indocyanine green (ICG) or low density lipoprotein (LDL) verified that the nanocomplex facilitated mouse embryonic stem cells to differentiate into functional and mature hepatocytes in vitro. When transplanted into mice model of CCl_4_-induced liver injury, the mouse embryonic stem cells treated with the functionalized nanocomposite were induced more robust differentiation of hepatocyte-like cells and significantly ameliorated liver injury and fibrosis. Compared with the other groups, the complex group prominently decreased the AST and ALT levels in the CCl_4_-injured mouse model (*P* < 0.05 relative to the growth factors alone group). The GF-PEI-MSN complex offered a sustained delivery of growth factors in CCl_4_-induced liver injury and led to reconstitution of the injured liver tissues after transplantation in vivo, thus conspicuously alleviating the liver injury [10]. In addition, HGF plays an important role in redox homeostasis through regulating the expression of SOD and GPX and reveals positive impacts on liver regeneration of ALF [159, 160]. Lin et al. synthesized HGF-loaded PLGA nanoparticles by using the W/O/W emulsion-solvent evaporation method. HGF-loaded PLGA showed superior therapeutic benefits on CCl_4_-induced ALF, such as enhancing the levels of SOD and GPX, decreasing the necrosis areas of liver tissues and reducing the AST and ALT levels [161].

### Nanozyme for Therapy of Acute Liver Failure

Nanozyme is one type of nanomaterials with enzyme-like characteristics [162, 163]. SOD and catalase mimics can detoxify various ROS, such as superoxide anion (O^2−^) and H_2_O_2_, which have been used in oxidative stress associated diseases [164–166]. Metalloporphyrin can imitate catalase, SOD, and other natural antioxidant enzymes [166]. For instance, manganese porphyrin as catalase and SOD mimics has been investigated in different studies [167]. Lately, pegylated manganese protoporphyrin, a water-soluble metalloporphyrin-based catalase mimic, was synthesized in Zhang’s work. Its therapeutic efficacy was also evaluated in APAP-related ALF model. Pegylated manganese protoporphyrin effectively removed H_2_O_2_ and reduced the liver-to-body weight ratio and serum ALT levels in vivo. It was convinced that pegylated manganese protoporphyrin could be a potent therapeutic agent for ALF [168].

Analogous to nanozyme, antioxidative nanobiomaterials can also mitigate APAP-induced hepatotoxicity and oxidative stress. Boonruamkaew et al. investigated a novel antioxidative nanoparticle, which was constructed via the crosslinking of the amphiphilic block copolymer, methoxy-poly(ethylene glycol)-b-poly[4-(2,2,6,6-tetramethylpiperidine-1-oxyl) aminomethylstyrene] [169]. On the side chain of the hydrophobic segment, the antioxidative nanoparticle possessed nitroxide radicals, which enabled the nanoparticle to obtain the capacity of scavenging ROS. The antioxidative nanomaterial inhibited the levels of ALT, AST, alkaline phosphatase (ALP), and O^2−^ and increased the amount of albumin of serum in APAP-associated ALF.

## Mechanism for Nanomedicine Targeting to the Liver in Acute Liver Failure Therapy

Approaches for nanoparticles delivery are generally divided into passive accumulation and active targeting (receptor-mediated targeting) [87, 170]. Passive targeting refers to that the physiological and anatomical features of the liver allow nanoparticles with specific sizes and surface properties to accumulate in the liver [170]. Active targeting makes use of specific ligands to bind to receptors on a certain type of liver cells, to achieve a selective drug/gene delivery to specific cells [171, 172]. Actually, these two targeting strategies usually complement each other. Cellular internalization of ligand-modified nanoparticles can be achieved only by relying on passive liver uptake mechanisms.

### Passive Targeting

The specific properties of nanoparticles such as size, hydrophilicity, and surface charge are the most fundamental factors that determine whether they have superior cell or tissue targeting capacity. Appropriate adjustments of the physicochemical features enable nanomedicines to enter liver cells in a passive-targeted manner, thereby increasing the accumulation of liver and reducing renal clearance and undesired distribution in other organs [24].

Particle size notably affects cell and tissue uptake. Liu et al. observed that nanoparticles smaller than 5 nm were rapidly cleared via renal filtration, resulting in low plaque accumulation [173]. In the liver, since the diameter of fenestrae in normal liver sinusoid endothelial cells is about 50–200 nm [174, 175], nanoscaled particles with diameters < 200 nm can pass through sinusoids [170]. This is beneficial for nanoparticles to evade capture by Kupffer cells and reach hepatocytes or hepatic stellate cells [24, 176]. Nanoparticles with diameters > 400 nm can diffuse through the sinusoid endothelial fenestrations by forced extrusion, presumably owing to transient interaction with the sinusoidal endothelial cells [177]. As a result, particles with larger size are more likely to be phagocytized by Kupffer cells [178], especially 200–1000 nm [179], even microbubbles of ultrasound imaging with the diameters of 1–10 µm [180].

Nanoparticles with hydrophobic surface are readily identified by mononuclear phagocyte system and primarily captured by spleen and liver [181]. Moreover, they are quickly removed from the systemic circulation [182]. By contrast, nanoscaled particles with hydrophilic surface are hardly taken up by the mononuclear phagocyte system. The nanoparticles with hydrophilic properties can enhance their permeation and retention effects, thereby increasing the accumulation in liver [87].

The surface charge is also a factor that affects the cellular uptake of nanoparticles. Positively charged particles prefer to be internalized by hepatocyte [182, 183], while Kupffer cells present a greater tendency to ingest nanoparticles with negative charges [4]. The proper cationic charge density of particles is necessary for greater membrane affinity and nucleic acid binding affinity [184, 185]. However, in the blood circulation, serum proteins with negative charges are possibly adsorbed on the surface of the nanoparticles. Furthermore, antibodies or complement proteins may also deposit on the surface, leading to poor serum stability [170]. The aggregates with the size over 200 nm and a larger negative surface charge will be eliminated by resident macrophages of the reticuloendothelial system in the liver, spleen, and bone marrow [170, 184, 185]. Therefore, it is important to achieve a balance between cell internalization and serum stability. PEG decoration on the nanoparticle surface is one of the common methods to improve the stability of particles in blood circulation, which covers positive charge on the surface of polyplexes and minimizes serum protein binding [186]. In addition, zwitterionic polymers with anionic and cationic end groups also show outstanding resistance to undiscovered immunogenicity and non-specific protein adsorption, which has been considered as a potential alternative of PEG [187].

After nanoparticles being administrated into blood circulation, the non-specific interactions between serum proteins and nanoparticles result in the formation of the protein corona which mediates the uptake of nontargeting nanoparticles in liver cells [24]. The physicochemical properties of nanoparticles determine the type of targeted hepatic cells. Non-targeted or unmodified nanomaterials are mainly absorbed by non-parenchymal cells in the liver, such as Kupffer cells, liver sinusoidal endothelial cells (LSECs), and hepatic stellate cells, predominantly capturing by Kupffer cells [112].

Kupffer cells are the resident macrophages of the liver, comprising ~ 35% of the non-parenchymal cells. Kupffer cells express scavenger receptors, mannose receptors, toll-like receptors, antibody receptors, and complement receptors, which facilitate the internalization of pathogens or foreign materials. Nanoparticles can be internalized through clathrin-mediated or caveolin-mediated endocytosis, macropinocytosis, and other endocytotic mode [188]. Similar as macrophages, LSECs possess phagocytic ability. Using scavenger receptors, mannose receptors, LSECs can directly take up materials from the bloodstream [112]. LSECs and Kupffer cells may compete for nanoparticles in the liver sinusoid [189]. Hepatic stellate cells distribute within the space of Disse which is a narrow space between LSECs and hepatocytes [22]. Nanomaterials that can pass through the sinusoidal fenestrae and not remove from Kupffer cells and LSECs may be taken up by hepatic stellate cells [112].

### Active Targeting

**Fig. 7:**
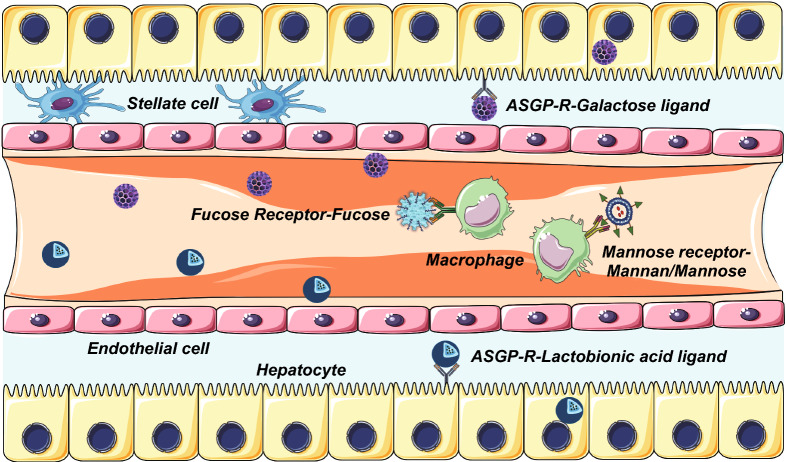
Schematic illustration depicting active targeting of hepatocytes and macrophages in ALF therapy

Nanoparticles functionalized with ligands are usually recognized by specific receptors of a particular cell or disease. The active targeting delivery nanosystems maximize the therapeutic effects of drugs and minimize the side effects of non-specific cellular uptake [170]. Ligand-mediated active targeting has been applied in therapy or diagnosis of liver diseases. Targeted cells include macrophages, hepatocytes, hepatic stellate cells, and endothelial cells. In the treatment of ALF, targeting macrophages (Kupffer cells) and hepatocytes are the most prevail therapeutic strategies (Fig. 7).

#### Targeting Hepatocytes

ASGP-R is a specific receptor, expressed on the membrane of hepatocytes near sinusoids [171]. The number of ASGP-R on each hepatocyte surface is between 100,000 and 500,000 [190]. ASGP-R possesses the innate binding affinity to galactose and N-acetyl-galactosamine residues. Its common target ligands are galactose, galactoside, galactosamine, lactobionic acid, asialofetuin, and sterylglucoside, which are usually exploited to modify nanomaterials for active targeting [170, 191]. Nanoparticles with the above ligands can be recognized by ASGP-R and taken by hepatocytes via clathrin-mediated endocytosis. Upon releasing the ligand, these receptors rapidly return to the cell membrane [171]. Taking advantage of the recognition ability of ASGP-R on hepatocytes, the ligands-functionalized nanomedicines have been widely employed in studies of liver-related diseases [171].

Galactose and lactobionic acid ligands are the most frequently used targeting modalities in targeted therapy of ALF. Due to the specific interaction between ASGP-R and galactose [192], galactosylated proteins or nanoparticles are able to be recognized by ASGP-R of hepatocytes, with a high affinity and a rapid internalization rate to hepatocytes. Jiang et al. constructed the galactose-modified cationic liposome to deliver Fas siRNA into hepatocytes, which effectively silenced Fas gene in ALF treatment [83]. In addition, lactobionic acid, an oligosaccharide aldonic acid, is also verified as the specific ligand of the hepatocyte [193]. Xiao et al. synthesized the IL-1Ra-lactosylated chitosan nanoparticles via coupling lactobionic acid with IL-1Ra chitosan nanoparticles to transport IL-1Ra. Consequently, the nanoparticles were successfully taken in hepatocytes through the interaction between ligand and receptor [82].

Besides, ASGP-R ligand-modified nanomaterials have been widely studied in the targeted treatment of liver cancer or hepatitis. Nanoparticles combined with galactoside or galactosamine are commonly used as targeted drug delivery systems for hepatocellular carcinoma [194–197]. In addition, Diez et al. applied asialofetuin ligand in interleukin-12 (IL-12) transfection for hepatocellular carcinoma therapy in vivo, in which the transfection activity of IL-12 in the liver was significantly improved [198]. Qi and co-workers synthesized soybean-derived sterylglucoside and polyethylene glycol modified hepatocyte-specific cationic liposome, which showed obvious inhibition in HBV replication in vitro [199]. Galactoside, galactosamine, asialofetuin, and sterylglucoside can be potential target ligands of ALF therapy.

#### Targeting Macrophages

Mannose receptor, a transmembrane protein, is overexpressed on the surface of macrophages or Kupffer cells [200]. d-mannose can be recognized by the mannose receptor [191]. The characteristic has been adopted to design macrophage-targeted nanoparticles. For instance, Zhang et al. applied carboxylated mannan-modified Se-PEI/TNF-α siRNA nanopolyplexes to bind with mannose receptor on macrophages for improving the cellular internalization rate of TNF-α siRNA [51]. Similarly, Higuchi and co-workers used mannosylated cationic liposomes for targeted delivery of NF-κB decoy to Kupffer cells [79].

Fucose receptors are uniquely expressed on Kupffer cells; therefore, fucose receptor-mediated delivery is also a potential way to target Kupffer cells [201, 202]. It was revealed that fucosylated protein was more easily to be phagocytized by macrophages [201]. Akao et al. utilized the fucose-modified dendrimer/α-cyclodextrin NF-κB decoy conjugates to efficiently inhibit the NF-κB expression of Kupffer cells in the liver [77].

Owing to the size and surface properties, nanomedicine can be enriched in the liver. Nanomaterials modified by targeting ligands are able to selectively target certain cells, significantly increasing the distribution of drugs and gene transfection. These endow nanomedicine with the advantages of improving therapeutic effect and reducing side effects.

## Nanobiomaterials as Imaging Agents for Acute Liver Failure Theranostics

Nanomaterials with optical or magnetic properties can be used as contrast agents for cells or tissues in ultrasound, X-ray, CT, or magnetic resonance imaging (MRI) imaging. They are usually utilized to label transplanted stem cells for assessing cell distribution or viability, or to mark the site of liver injury for diagnosis of ALF or the evaluation of curative effect. Additionally, these nanomaterials are usually co-loaded with different drugs and serve as theranostic agents for ALF therapy.

### Fluorescence Imaging

Stem cell transplantation is considered as an alternative therapy to liver transplantation in ALF. It exerts promising therapeutic effects for patients in clinical applications [6–8]. Although stem cell transplantation possesses considerable potential in promoting liver regeneration, the lack of technology for long-time tracing biological distribution and behavior of transplanted cells limit the development of stem cell-based ALF therapy [9, 203, 204]. Studies have demonstrated that the combination of nanotechnology and stem cell therapy is beneficial to make up for this deficiency [9]. Yukawa et al. investigated quantum dots (QDs) labeled with octa-arginine peptide (R8) to track the transplanted cells in vivo [203]. QDs were applied in fluorescence imaging of adipose tissue-derived stem cells (ASCs) to trace the transplanted stem cells in the liver, thereby visualizing organ-specific accumulation of ASCs in mice model of CCl_4_-induced ALF. Moreover, ASC transplantation was combined with heparin, finding that the accumulation rate of ASCs in the liver increased to nearly 30%. The fluorescence imaging also assisted in confirming the efficiency of heparin in ALF therapy.

**Fig. 8:**
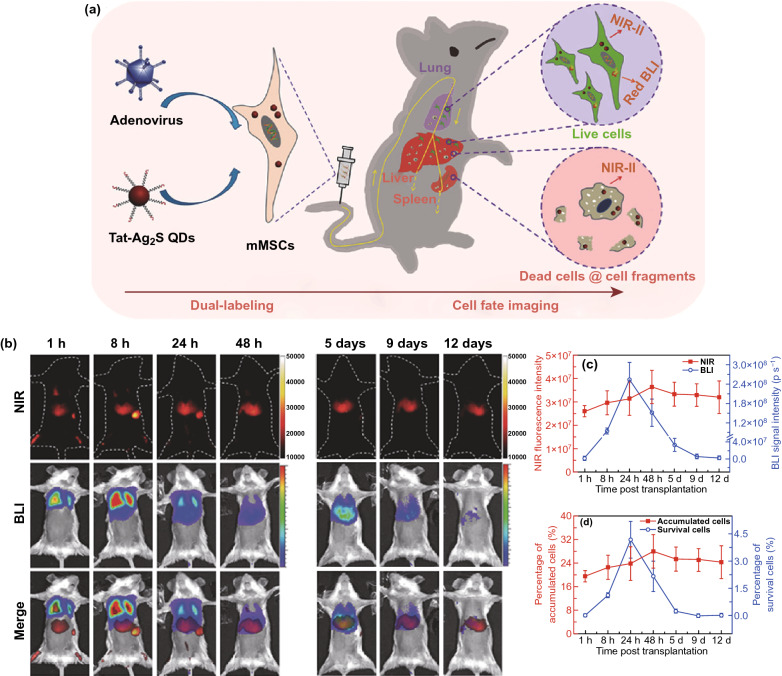
**a** Schematic representation depicting dual-labeling for the transplanted MSCs. Dual-labeling: endogenous red bioluminescence imaging (BLI) combined with exogenous near-infrared fluorescence imaging of second window (NIR-II) by using red-emitting firefly luciferase and Tat-Ag_2_S QDs. **b** Tracking images of transplanted MSCs in ALF mice (NIR-II fluorescence, BLI and merged images). **c** Quantitative analyses about the number of accumulated or survived cells in liver through the total NIR fluorescence intensities of near-infrared fluorescence imaging and the total photon flux of BLI. **d** Quantitative analyses of percentages of accumulated and survival cells in the transplanted MSCs in liver. Reprinted with permission from Ref. [204]

Afterward, Chen and co-workers investigated a dual-labeling method, in which the endogenous red bioluminescence imaging was combined with exogenous near-infrared fluorescence imaging in the second window (NIR-II) [204], to monitor the survival rate and the cell clearance of transplanted stem cells in ALF treatment. The NIR-II fluorescence of exogenous Tat-Ag_2_S QDs was used for locating and quantifying transplanted MSCs (both dead and living cells). Meantime, the endogenous red-emitting firefly luciferase was employed to mark the living MSCs. Therefore, the colocalization indicated the transplanted living MSCs. This facile imaging method enabled direct visualization of the whole-body distribution, translocation, cell clearance, and viability of transplanted MSCs in the ALF mouse model by the overlapped red bioluminescence imaging and NIR-II fluorescence signals (Fig. 8).

### Magnetic Resonance Imaging

Magnetic nanomaterials are also used for cell labeling, serving as contrasting agents in cellular MRI. Superparamagnetic iron oxide particles (SPIOs) are one of the widely used contrast-enhancing agents in cellular MRI due to their large magnetic moment, which possess the greatest change in signal intensity per unit of metal. Iron oxide-labeled cells are detected as low signal intensity areas on *T*_2_- or *T*_2_-weighted imaging of MRI [205]. SPIO coated with dextran is an FDA-approved iron oxide formulation that has been extensively utilized in biomedical studies. In Puppi’s work, hepatocytes were marked with SPIOs and protamine sulfate in vitro, which increased cellular iron uptake and did not impair metabolic function or viability of hepatocyte [206]. Labeled hepatocytes were transplanted in a rat model of ALF by intrasplenic transplantation. Six days post-transplantation, SPIOs were detected in the liver of the surviving rat on *T*_2_-weighted imaging of MRI. Using SPIOs to label cells showed the potential for assessing the biodistribution of hepatocytes in the early phases after transplantation. Nevertheless, its defect that SPIOs-labeled hepatocytes were cleared by Kupffer or endothelial cells impeded the application of long-term detection in transplanted cells. Besides, magnetic nanomaterials as MRI contrast agents are applied in the diagnosis of acute liver injury. Xu et al. synthesized hydroxyapatite-Fe_3_O_4_ nanoworms. Hydroxyapatite as a core was coated with an anionic polymer, poly(sodium-p-styrenesulfonate). Fe_3_O_4_ NPs were adsorbed to the hydroxyapatite nanocrystals. Finally, chitosan and sodium alginate were coated on the surface to enhance the biocompatibility of the nanoworms. The study revealed that the nanoworms had outstanding MRI diagnostic ability for acute liver injury. The contrast to noise ratio of impaired liver tissues was enhanced from 3.71 to 5.39 by using the nanocomposites. The nanoworm was a noninvasive diagnostic strategy for acute liver injury, which was conducive to injury grading [207].

### Computed Tomography Imaging

**Fig. 9:**
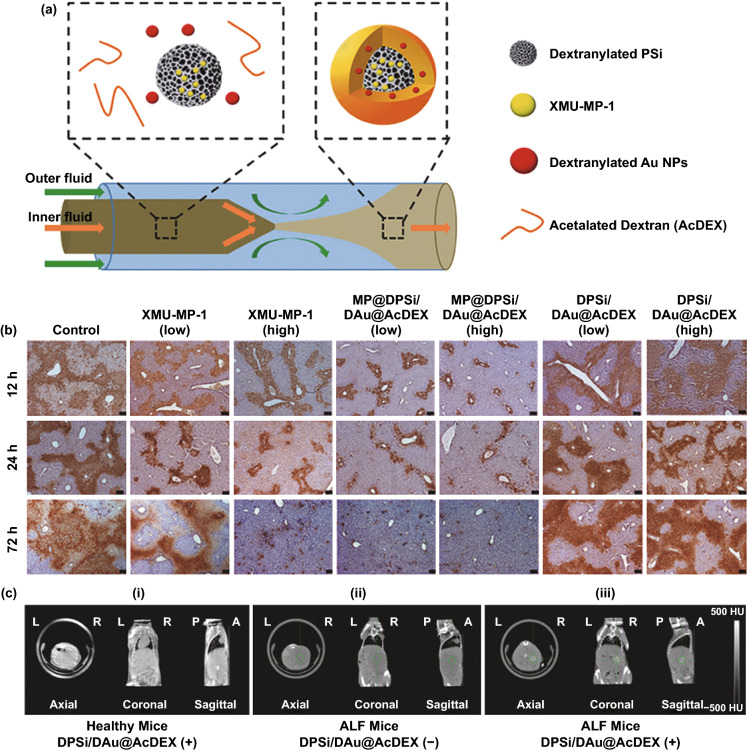
**a** Preparation of the nanocomposite composed of dextranylated porous silicon, XMU-MP-1, dextranylated Au NPs, and acetalated dextran (MP@DPSi/DAu@AcDEX) by microfluidics. **b** TUNEL staining of liver tissues in APAP-intoxicated mice at 12, 24, and 72 h post-APAP administration. (control: 15% Solutol^®^ HS 15; XMU-MP-1: 0.1 mg kg^−1^ (low), 0.5 mg kg^−1^ (high), dispersed in 15% Solutol^®^ HS 15; MP@DPSi/DAu@AcDEX: XMU-MP-1: 0.1 mg kg^−1^ (low), 0.5 mg kg^−1^(high); DPSi/DAu@AcDEX: (without XMU-MP-1), the concentration is corresponding with MP@DPSi/DAu@AcDEX (twice daily i.v. injection 2.5 h after APAP challenge). **c** CT images of mice after DPSi/DAu@AcDEX treatment. i) healthy mice, i.v. injection of DPSi/DAu@AcDEX, ii) ALF mice, without i.v. injection of DPSi/DAu@AcDEX, iii) ALF mice, i.v. injection of DPSi/DAu@AcDEX; (DAu: 20 mg kg^−1^, DPSi/DAu@AcDEX: 200 µL). Reprinted with permission from Ref. [173]

Except for applications of tracking transplanted cells and diagnosis in ALF, the stimuli-responsive nanomaterials with outstanding optical or magnetic properties can be applied as the multifunctional nanomedicines with diverse applications, such as targeted therapy, diagnosis, imaging, and monitoring of the treatment process [78, 171]. A novel nanocomposite composed of porous silicon, gold nanoparticles, and surface-coated acetalated dextran, was developed by microfluidics-assisted nanoprecipitation method [173]. This system was a multifunctional platform that could encapsulate and transmit drug, as well as increase the CT signal in ALF theranostics. 4-((5,10-dimethyl-6-oxo-6,10-dihydro-5H-pyrimido[5,4-b]thieno[3,2-e] [1, 4] diazepin-2-yl)amino) benzenesulfonamide (XMU-MP-1) [208], a selective inhibitor of kinases MST1 and MST2 (MST1/2), displayed remarkable function in augmenting liver repair and regeneration in acute or chronic liver injury mouse models. XMU-MP-1 was encapsulated and delivered by the above-mentioned nanocomposite to facilitate liver regeneration in ALF. In TUNEL staining of liver tissue, the nanocomposite showed a distinct alleviation even reversion of liver injury than free drug. Simultaneously, as the nanocomposite was mainly ingested by macrophages that locally accumulated in the damaged area, gold nanoparticles in the nanocomposite as contrasting agents of CT imaging enhanced the local signal in the lesion areas of liver. This method was expected to be an early diagnosis method for ALF (Fig. 9).

### Ultrasound Imaging

**Fig. 10:**
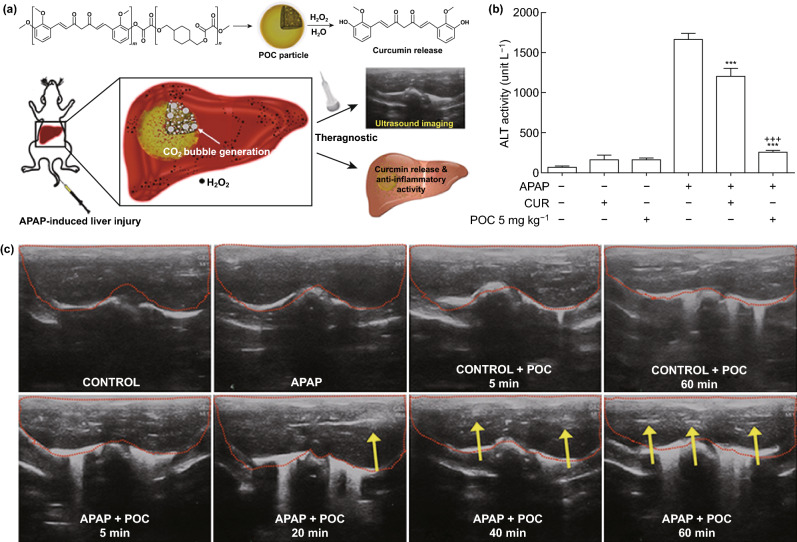
**a** Schematic illustration of the synthesis of POC particle (poly(oxalate-co-curcumin)) as a theranostic agent for ALF. **b** Therapeutic effects of POC particle in APAP-induced ALF: the serum levels of ALT in different groups (CUR (Curcumin 0.75 mg kg^−1^) or POC particles 5 mg kg^−1^ through tail vein injection. After 1 h, APAP-induced ALF was established by the intraperitoneal injection of 400 μL APAP solution 22.5 mg mL^−1^) (mean ± SD (*n* = 3), ***P < 0.001 relative to APAP-treated group, ^†††^*P *< 0.001 relative to CUR). **c** Ultrasound images of livers (red: the liver contour; yellow: the echogenic POC particles). Reprinted with permission from Ref. [14]

Ultrasound imaging is another widely used diagnostic method in the clinic. Because of easy detection, low cost and no radiation hazard, it has been used as a screening method for liver disease. Conventional ultrasound contrast agents are microbubbles composed of a shell material of lipids and proteins and a gas core, whose diameter is 1–8 μm [209–211]. However, there exist undesired imaging defects, such as poor blood vessel stability, short echo persistence, and difficulty in penetrating surrounding tissues [212]. By contrast, pathological stimulus-triggered echogenic nanoparticles can generate bubbles at injured sites, which penetrate from blood vessels and accumulate in targeted tissues to specifically enhance ultrasound imaging signals [213]. For instance, Go et al. reported an acid-triggered echogenic ketalized maltodextrin nanoparticle, a pathological stimulus-activatable nanoplatform that could simultaneously deliver therapeutic and imaging agents to the acidic inflammatory site [214]. It served as a targeted therapeutic agent and ultrasound contrast agent in APAP-induced ALF. In the acidic environment of the inflammatory regions, ketalized maltodextrin nanoparticles generated CO_2_ bubbles due to acid-triggered decarboxylation of carbonate, which enhanced the ultrasound signal in the damaged liver. In addition to ultrasound imaging agents, ketalized maltodextrin served as the targeted drug delivery system of silymarin. Silymarin had powerful antioxidant and anti-inflammatory activities, and hepatoprotection ability. Thus, the nanocomposite remarkably reduced TNF-α expression and ALT level of APAP-intoxicated mice in a dose-dependent manner. Analogous to ketalized maltodextrin nanoparticles, hydrogen peroxide-activatable peroxalate polymer can produce CO_2_ in a H_2_O_2_-induced manner. It was reported that the peroxalate polymers could act as both the ultrasound imaging agent for H_2_O_2_-related inflammatory diseases [210, 211], and the backbone of polymeric prodrugs for biologically active compounds [215, 216]. Curcumin has potent antioxidant and ROS scavenging pharmacological effects because of the capacity of providing hydrogen atoms [217]. However, poor stability and solubility limit its application in inflammatory diseases [218, 219]. Berwin Singh et al. designed and synthesized H_2_O_2_-responsive poly(oxalate-co-curcumin) (POC) nanoparticles, in which curcumin was covalently bound to the polymer framework via peroxalate ester [14]. POC showed excellent anti-inflammatory and highly potent antioxidant activity. Under oxidative stress, the reaction between H_2_O_2_ and POC triggered the release of curcumin and the production of nano or microbubbles of CO_2_. In APAP-intoxicated mice, POC significantly reduced the ALT level than the free curcumin group (*P *< 0.001 relative to the free curcumin group). The generated CO_2_ enhanced the ultrasound signal of APAP-intoxicated liver and endowed the nanoparticle with the ability of ultrasound contrast agents (Fig. 10). POC held great potential as theranostic agents for ALF.

Optical or magnetic nanomaterials, and stimuli-responsive echogenic nanoparticles (e.g., QDs, SPIOs, iron oxide, Au NPs, ketalized maltodextrin nanoparticles, poly(oxalate-co-curcumin) nanoparticles) have emerged as potential candidate systems for tracing the transplanted MSCs or ALF theranostics in current research. Nanotechnology for stem cell labeling and tracking provides an accurate monitoring of transplanted stem cell in animal models. Moreover, the multifunctional nanoparticles hold the promise of more precisely targeted treatment and detection and play an important role in therapeutic decision-making. At present, Au NPs [13, 220] and SPIOs [221–223] have been widely applied in vivo and in several clinical applications. Previous research reported that iron oxide-based nanomaterials were slowly degraded in the mononuclear phagocyte system and combined with hemosiderin or ferritin [224, 225]. However, some research reported that inorganic nanomaterials were relatively stable and able to persist in the liver even 6–15 months after administration, such as gold [226, 227], iron oxide [228], and iron oxide-coated gold nanoparticles [229]. Notably, gold nanostructures were not toxic in the long term. The gold nanoparticles were not found to exert any adverse impact on the animals during the time frame (a few weeks to a year), in spite of major differences in biodistribution [230]. Therefore, more studies about the fate and elimination pathways of nanomaterials in vivo are needed to be performed in the treatment of ALF, which are main barriers for the further clinical application of these nanomaterials.

## Challenges and Perspectives

We have summarized the recent advances of nanomaterials in the treatment of ALF (Table 1). As our understanding of ALF and nanomaterials deepens, potential applications of nanomaterials in the therapy and imaging of ALF broaden. At present, etiological treatment, hepatoprotective drugs, albumin supplementation, serum bilirubin reduction, plasmapheresis, treatment and prevention of complications, stem cell transplantation and support therapy are mainly used for ALF treatment in clinic [231]. However, none of these treatments can completely cure ALF, and there is no specific medicine for ALF treatment. Liver transplantation is the only curative therapy so far, nevertheless, expensive costs, lack of donor livers, and complications associated with immunosuppression limit its application [3]. Free therapeutic agents (antioxidant or anti-inflammatory drugs, nucleic acids, and cytokines) delivered by systemic administration, only showed limited efficacy in clinical trials [15, 16, 141, 232]. This unsatisfactory therapeutic effect has been attributed to premature clearance, poor blood system stability, difficulty in maintaining the effective doses for a long period, low drug accumulation at the injured site, and adverse effects result from supplementation overdose [17, 18, 141, 233, 234]. Nanomaterials possess distinct advantages in ALF therapy owing to their intrinsic properties. On the one side, nanobiomaterials delivery systems offer many benefits over the biologic agents alone. Drugs encapsulated in nanocarriers are generally more stable in blood circulation with higher bioavailability than the free drug (such as silymarin [214], andrographolide [81], naringenin [76], and curcumin [14]). Nano-formulations allow more anti-inflammatory/antioxidant drugs with poor water-solubility and short half-life to be used for ALF treatment. Naked nucleic acids are readily degraded by nucleases and entrapped by endosome/lysosome. Non-viral gene delivery platforms based on nanobiomaterials exhibit the ability of nucleic acids protection and excellent endosomal escape capability. Nanobiomaterials delivery systems can improve the stability of cytokines and avoid the treatment of high-dose injection. In addition, nanobiomaterials increase the accumulation of therapeutic agents in the diseased liver and enable more effective targeting of the liver or specific liver cells, thereby limiting undesired organ toxicities and reducing off-target adverse effects. Stimuli-responsive nanomaterials can perform spatiotemporal control that therapeutic cargo keeps inactive until it accumulates in target sites or cells. On the other side, nanobiomaterials have their own therapeutic and imaging functions. For instance, H_2_O_2_-responsive polyoxalate [14], the antioxidant polymeric prodrug microparticles: poly(vanillyl alcohol-co-oxalate) (PVAX) [12], ROS-responsive poly(ethylene glycol)-b-poly(propylene sulfide) [74], these antioxidant/anti-inflammatory drug delivery systems show synergistic therapeutic effects in ALF therapy. Magnetic or optical nanomaterials such as SPIOs [205], Au NPs [173], or QDs [203] are utilized for ALF diagnosis, imaging assessment of therapeutic effects, or transplanted stem cell labeling, even multifunctional theranostic platform. Magnetic or optical nanomaterials promote the development of theranostic nanomedicine for ALF by combining diagnosis with specific targeted therapy. Therefore, therapeutic agents in combination with nanomaterial offers advanced targeted nanodrug, even multifunctional theranostic platform. Nanomedicine holds excellent prospects in the area of ALF theranostics. Despite the above-mentioned progress that has been made in the treatment of ALF, nanomedicine in this field is still in its infancy. There are still some challenges to be addressed.

1. More consideration should be given to the biocompatibility and biodegradability of nanomedicines in ALF therapy. Nanomaterials with high biocompatibility and biodegradability can avoid the liver burden and decrease side effects to other organs. ALF usually causes systemic inflammation, even systemic inflammatory response syndrome and multiorgan failure. Any drug that damages the liver or other organs will lead to the progress of the disease. Therefore, nanomaterials with low hepatotoxicity are crucial for the therapy of ALF to avoid further liver dysfunction.
2. Further exploration and systematic evaluation of long-time cytotoxicity, pharmacokinetics, distribution, metabolism, and related immune response of nanomedicine are of vital importance. According to the current research, although nanobiomaterials show negligible cytotoxicity to cells or organs in a short time range, the potential safety effects and long-time cytotoxicity in vivo are of great concern. There is limited knowledge about the pharmacokinetics of nanoparticles, such as absorption, secretion, distribution, and metabolism processes in vivo. Nanoparticles may accumulate in lung, liver, spleen, or kidney [235, 236], and off-target effects of them may lead to the occurrence of acute liver or kidney injury and inflammatory response [237, 238]. To assess toxicity, Moustafa et al. studied the long-term effects of gold nanorods (AuNRs) in vivo. It was demonstrated that most of AuNRs accumulated in the liver and spleen for up to 15 months, and a very small portion of AuNRs was excreted from the feces of mice. Although there is no obvious histopathological abnormality in liver and spleen 15 months after AuNRs injection [226], whether the long-term accumulation of nanomaterials will increase the occurrence of immune-related reactions need further investigation. Notably, side effects and systemic toxicity not only result from nanomaterials themselves, but also from the chemicals or solvents involved in the synthesis process [239].
3. The design and development of nanomedicines with highly targeting ability toward liver cells will be one of the greatest challenges to be tackled. For one thing, more nanomaterials equipped with selective ligands are required to be designed, particularly in the targeting of the hepatocyte. The receptors and ligands targeting hepatocytes and Kupffer cells also need to be further explored. For another, due to the overlapping expression of receptors in different liver cells, for instance, both mannose receptors and scavenger receptors are on the surface of Kupffer cells and liver sinusoidal endothelial cells [240]. Therefore, targeted nanomedicines decorated with exclusive specific ligand will be the future direction of development. Finally, ligands and receptors of other liver cells, such as hepatic stellate cells and endothelial cells, still need to be further investigated and developed.
4. The transmembrane transport pathway is closely related to nucleic acid intracellular fate and it ultimately determines the therapeutic efficiency [241]. It is necessary to explore the transmembrane trafficking mechanism mediated by a variety of nanomaterials. Non-viral vector gene delivery-based on nanobiomaterials, including cationic polymers (PEI [51], PAMAM [80]), and liposomes, enters cell mainly through membrane fusion or endocytic mechanism [141, 242]. Nanobiomaterials entered cells via endocytosis are often entrapped in endosome/lysosome [243], thus increasing the possibility of nucleic acids degradation and off-target effects [244]. However, membrane fusion may avoid the endosome/lysosome entrapment [141]. Although polyamine polymers and dendrimers can facilitate endosome/lysosome escape of nucleic acids by “proton sponge effect” [141, 245], cationic polymers are readily accumulated in the lung vascular tree after intravenous injection [78]. Hence, the development of nanobiomaterials mediated by membrane fusion should be focused on to achieve high transfection efficiency.
5. Nanotechnology is an assisted therapy strategy for stem cell-based therapy. For one thing, more nanobiomaterials for labeling stem cell with high sensitivity, spatial and temporal resolution capabilities need to be developed in ALF therapy [9]. For another, nanobiomaterial-assisted stem cell therapy has been applied in cardiac repair, recovery of vision loss and bone regeneration [246–249]. Similar studies can be carried out in the treatment of ALF.
6. Theranostic nanomedicines have become a promising therapeutic paradigm by combining diagnosis with specific targeted therapy. The development of multifunctional nanoparticles with advanced imaging, diagnosis, drug delivery, and monitoring of therapeutic response potential will be a hot topic for future research. So far, some multifunctional nanohybrids with ultrasound, CT or MRI imaging, diagnostic, and therapeutic functions have been investigated in ALF. However, the accuracy of diagnosis and efficacy of treatment in the comprehensive platform need to be further improved. In addition, the development and application of nanomaterial-based contrast agents in ALF diagnosis and efficacy evaluation have great clinical significance. The elimination mechanism of optical or magnetic nanomaterials, such as Au NPs, inorganic nonmetallic contrast agents, iron oxide-based materials, is critical to the clinical translation of nanomaterial-based contrast agents. The indications and contraindications of nanomaterial contrast agents in clinical application are based on the clearance pathways. Nevertheless, there is no data to reveal whether optical or magnetic nanomaterials are excreted through the renal pathway via the urine or are eliminated from the body via biliary and fecal mechanisms. How long does it take to completely remove these materials in vivo?
7. According to the aforementioned studies, most of nanomedicines alleviate liver damage through anti-inflammatory or antioxidant effects, nevertheless, hitherto little information about the specific mechanism of nanomedicines has been elaborately explored. The mechanism of nanomaterials on ALF therapy should be deeply investigated to provide instructive insights for the design and improvement of nanomedicines.
8. In current experimental studies, experimental models are generally confined to APAP, CCl_4_- or LPS/D-GalN-induced ALF animal models, however, ALF related to viruses, autoimmune diseases or other genetic factors remains uninvolved. It is necessary to employ nanomedicines into the therapy of ALF with the above pathogenesis, particularly HBV associated ALF.
9. Therapeutic efficiency evaluation of nanobiomaterials in vivo is incomplete. Most of research use AST and ALT levels, inflammatory factor levels (such as TNF-α, IL-1β, and IL-6), and animal survival rates to evaluate treatment outcomes. More indicators of hepatic regeneration, such as coagulation function, the serum bilirubin and albumin levels are needed to be supplemented in future studies.

**Table 1:** Nanoparticles for theranostics of ALF

Nanoparticle	Drug/gene/other delivery	Targeted cell	Receptor-ligand	Application	References
Silymarin-loaded ketalized maltodextrin	Silymarin	–	–	H_2_O_2_-responsive theranostic agent Ultrasound imaging agent, drug carrier, anti-inflammatory	[214]
DPSi/DAu@AcDEX^*a*^	XMU-MP-1	Macrophages	–	Theranostic agent CT imaging agent, drug carrier, alleviate liver damage	[173]
POC	Curcumin	–	–	H_2_O_2_-responsive theranostic agent Ultrasound imaging agent, drug carrier, antioxidant	[14]
HAP-ION nanoworms^*b*^	–	–	–	Diagnosis MRI imaging agent	[207]
QDs + R8	–	Labeling transplanted ASCs	–	Fluorescence imaging agent Tracking transplanted stem cells	[203]
NIR-II (Ag_2_S QDs)	–	Labeling transplanted MSCs	–	Fluorescence imaging agent Tracking transplanted stem cells	[204]
SPIO + PS^*c*^	–	Labeling transplanted hepatocytes	–	MRI imaging agent Tracking transplanted hepatocytes	[206]
PVAX	Manganese porphyrin	Macrophages	–	Therapeutic agent Drug carrier, antioxidant	[12]
PAOX	PTX	Macrophages	–	Therapeutic agent Drug carrier, anti-inflammatory	[75]
NARN^*d*^	Naringenin	–	–	Therapeutic agent Drug carrier, antioxidant, anti-apoptosis	[76]
IL-1Ra chitosan nanoparticles	IL-1R antagonist	Hepatocytes	(ASGP-R)- Lactobionic acid	Targeted therapeutic agent Drug carrier, anti-inflammatory, promote hepatocyte proliferation	[82]
Hep-AGnp^*e*^	Andrographolide	–	–	Therapeutic agent Drug carrier, hepatoprotective effect, antioxidant	[81]
mPEG-b-PPS^*f*^	Melatonin	–	–	ROS-responsive therapeutic agent Drug carrier, anti-inflammatory, antioxidant	[74]
Man-liposome/NF-κB decoy	NF-κB decoy	Kupffer cells	Mannose receptor-Mannose	Targeted therapeutic agent Gene carrier, anti-inflammatory	[79]
Fuc-S-α-CDE^*g*^/NF-κB decoy	NF-κB decoy	Kupffer cells	Fucose receptor-Fucose	Targeted therapeutic agent Gene carrier, anti-inflammatory	[77]
PPABLG HNPs	TNF-α siRNA	Kupffer cells	–	Therapeutic agent siRNA carrier, anti-inflammatory	[78]
Man-COOH/Se-PEI/siTNF-α	TNF-α siRNA	Macrophages	Mannose receptor- Man-COOH	Targeted, ROS-responsive therapeutic agent siRNA carrier, anti-inflammatory	[51]
Gal-LipoNP Fas siRNA	Fas siRNA	Hepatocytes	(ASGP-R)- Galactose	Targeted therapeutic agent siRNA carrier, anti-inflammatory, anti-apoptosis	[83]
PDPIA	IL-22 gene	Hepatocytes	–	Targeted therapeutic agent Gene carrier, anti-inflammatory, hepatocyte regeneration	[80]
CHC/PU-PEI-miR122	MicroRNA122	–	–	Therapeutic agent MicroRNA carrier, promoting stem cell hepatic-specific differentiation	[84]
PEI-MSNs^*h*^	HGF, aFGF, activin A	–	–	Therapeutic agent Growth factors carrier, promoting stem cell hepatic-specific differentiation	[10]
MRIN	MSC-conditioned media	–	–	Therapeutic agent Growth factors carrier, anti-inflammatory, anti-apoptosis, promoting liver regeneration	[85]
Pegylated manganese protoporphyrin	catalase mimics	–	–	Therapeutic agent Catalase mimic carrier, anti-inflammatory, antioxidant	[168]

^a^DPSi/DAu@AcDEX: dextranylated PSi/dextranylated gold nanoparticles@acetalated dextran; ^b^HAP-ION nanoworms: hydroxyapatite-Fe_3_O_4_ worm-shaped nanocomposites; ^c^PS: protamine sulfate; ^d^NARN: naringenin-loaded nanoparticles; ^e^Hep-AGnp: heparin-functionalized andrographolide nanoparticle; ^f^mPEG-b-PPS: methoxy-poly(ethylene glycol)-b-poly(propylene sulfide); ^g^Fuc-S-α-CDE: fucose-appended dendrimer conjugate with α-cyclodextrin; ^h^PEI-MSNs: polyethyleneimine-modified mesoporous silica nanoparticles

## Conclusion

We have highlighted the progress made in nanobiomaterials for ALF therapy. Diverse types of nanobiomaterials, including cationic polymers and polypeptides, liposomes, inorganic nanocarriers, and nanozymes, have been used for the delivery of traditional biologic drugs, showing great superiorities in improving pharmacological properties, blood system stability or targeting ability and reducing side effect. During the last few years, multifunctional nanobiomaterials with imaging, diagnostic and therapeutic ability have gradually become the research trend in the treatment of ALF, providing a novel strategy for the theranostics of ALF. Despite the outstanding developments that have been made in recent years, there is still a long way for advancing future research. This field is highly dynamic and has broad application prospects in the future. Future breakthroughs in this field will bring about a new wave of original nanobiomaterials in the treatment of ALF by overcoming the above-mentioned potential challenges.
